# *FLOURY ENDOSPERM 6* mutations enhance the sugary phenotype caused by the loss of ISOAMYLASE1 in barley

**DOI:** 10.1007/s00122-023-04339-5

**Published:** 2023-04-03

**Authors:** Ryo Matsushima, Hiroshi Hisano, Ivan Galis, Satoko Miura, Naoko Crofts, Yuto Takenaka, Naoko F. Oitome, Takeshi Ishimizu, Naoko Fujita, Kazuhiro Sato

**Affiliations:** 1 Institute of Plant Science and Resources, Okayama University, 2-20-1 Chuo, Kurashiki, Okayama 710-0046 Japan; 2 College of Life Sciences, Ritsumeikan University, Kusatsu, Shiga 525-8577 Japan; 3 Department of Biological Production, Akita Prefectural University, Akita, 010-0195 Japan

## Abstract

**Key message:**

Barley double mutants in two genes involved in starch granule morphology, *HvFLO6* and *HvISA1*, had impaired starch accumulation and higher grain sugar levels than either single mutant.

**Abstract:**

Starch is a biologically and commercially important glucose polymer synthesized by plants as semicrystalline starch granules (SGs). Because SG morphology affects starch properties, mutants with altered SG morphology may be useful in breeding crops with desirable starch properties, including potentially novel properties. In this study, we employed a simple screen for mutants with altered SG morphology in barley (*Hordeum vulgare*). We isolated mutants that formed compound SGs together with the normal simple SGs in the endosperm and found that they were allelic mutants of the starch biosynthesis genes* ISOAMYLASE1 (HvISA1)* and* FLOURY ENDOSPERM *6 (*HvFLO*6), encoding starch debranching enzyme and CARBOHYDRATE-BINDING MODULE 48-containing protein, respectively. We generated the *hvflo6 hvisa1* double mutant and showed that it had significantly reduced starch biosynthesis and developed shrunken grains. In contrast to starch, soluble α-glucan, phytoglycogen, and sugars accumulated to higher levels in the double mutant than in the single mutants. In addition, the double mutants showed defects in SG morphology in the endosperm and in the pollen. This novel genetic interaction suggests that* hvflo6* acts as an enhancer of the sugary phenotype caused by* hvisa1* mutation.

**Supplementary Information:**

The online version contains supplementary material available at 10.1007/s00122-023-04339-5.

## Introduction

Starch is a plant-derived glucose polymer that is widely used in both food and industrial applications. Due to its water-insolubility and osmotic inactivity, it is an ideal molecule for long-term storage in seeds, grains, and roots. In such storage organs, starch is synthesized as semicrystalline starch granules (SGs) in specialized plastids known as amyloplasts. SGs differ morphologically among plant species and are classified as compound or simple SGs (Tateoka 1962; Matsushima et al. 2013; Chen et al. 2021). Compound SGs are formed from assemblies of smaller starch particles, whereas simple SGs are single starch particles. Rice (*Oryza sativa*) endosperm has compound SGs typically 10–20 μm in diameter and composed of individual sharp-edged polyhedral smaller starch particles with a typical diameter of 3–8 μm (Matsushima et al. 2010). Barley (*Hordeum vulgare*) and wheat (*Triticum aestivum*) endosperm has two types of simple SGs, the smaller B-type and the larger A-type, which coexist in a single cell (Matsushima et al. 2013), a storage type called bimodal simple SGs. A-type granules form in amyloplasts during early grain development, and B-type granules initiate later within the amyloplasts that already contain A-type granules (Langeveld et al. 2000; Matsushima and Hisano 2019).

SG size and morphology are affected by the specific enzymes involved in the biosynthesis of amylopectin, the major component of starch that is composed of α-1,4-linked, α-1,6-branched chains (Zeeman et al. 2010; Smith and Zeeman 2020). Amylopectin biosynthesis begins with the enzyme ADP-glucose pyrophosphorylase, which produces ADP-glucose, the glycosyl donor for the elongation of glucose chains (Figueroa et al. 2022). Three additional reactions are involved in amylopectin biosynthesis: formation of α-1,4-glycosidic bonds by starch synthases (SSs) to elongate glucose chains, creation of α-1,6-glycosidic bonds by branching enzymes (BEs) to create branches, and removal of branches by starch debranching enzymes (DBEs) (Nakamura 2002; Smith and Zeeman 2020). Plants have multiple isozymes of each of these enzymes, and mutations of some of these isozymes dramatically affect SG morphology in the endosperm. For example, a mutant of tetraploid wheat (*Triticum turgidum*) that is defective in both *Starch synthase 4* homoeologs develops compound SGs instead of simple SGs (Hawkins et al. 2021). DBEs trim misplaced branches of amylopectin by hydrolyzing α-1,6 linkages during amylopectin biosynthesis. Without such trimming, misplaced or excess branches can prevent the formation of the semicrystalline matrix of amylopectin. Deficiency of one of the DBEs, ISOAMYLASE1 (ISA1), results in the accumulation of the water-soluble α-glucan, phytoglycogen in rice, maize (*Zea mays*) and barley (Pan and Nelson 1984; Nakamura et al. 1997; Burton et al. 2002). Phytoglycogen is extensively branched with short glucan chains and has a much smaller molecular weight than amylopectin. The barley mutants *Risø17* and *Notch-2* have mutations in the barley *ISA1* gene (*HvISA1*) (Burton et al. 2002). Both mutants develop abnormal compound SGs in the endosperm (Burton et al. 2002).

Recently, several non-catalytic proteins have been identified to directly bind to starch biosynthetic enzymes, supporting their functions. These proteins possess conserved carbohydrate-binding modules 48 (CBM48) and facilitate the binding of starch biosynthetic enzymes to polysaccharides (Peng et al. 2014; Seung et al. 2015, 2017). *Arabidopsis thaliana PROTEIN TARGETING TO STARCH1* (*PTST1*) and *PTST2*, and rice *FLOURY ENDOSPERM 6* (*FLO6*) are well-characterized CBM48-containing proteins. These proteins bind to the starch biosynthetic enzymes and bring them into close contact with starch or maltooligosaccharides. The barley *Franubet* mutant (also called *hvflo6*) has a genetic lesion in *HvFLO6,* an ortholog of rice *FLO6* (Saito et al. 2018). The endosperm of this mutant does not have the bimodal simple SGs typical of barley, but instead contains a mixture of irregular simple SGs and compound SGs (DeHaas and Goering 1983; Chung 2001; Suh et al. 2004).

Mutants with altered SG morphology often show novel starch properties including altered digestibility, gelatinization temperature, amylose content, and amylopectin structure (Nakamura 2018; Chen et al. 2021). Understanding the causal genes of these mutations and their interactions is important for basic biology and industrial applications related to starch. We previously developed a rapid simple method to observe rice SGs and used this method for genetic screening of rice mutants with altered SG morphology (Matsushima et al. 2010, 2014, 2016). Unlike other procedures, this method does not require chemical fixation and resin embedding of samples, making it ideal for screening SG mutants in large cereal populations.

The barley endosperm develops bimodal simple SGs, which are only found in a few genera within the Poaceae family (Matsushima et al. 2013). To improve our understanding of the mechanisms that determine SG morphology in barley, further research is necessary, particularly regarding the genetic interactions between mutations of starch-related genes. Because existing barley mutants have been isolated from diverse genetic backgrounds, accurate evaluation of the genetic interactions is challenging due to the mixture effects of different genomes after crossing. Therefore, newly isolation of SG mutants from the same background of barley could be helpful. In this study, we used the rapid simple observation method to screen for SG mutants in barley using grains of a mutagenized population derived from the Japanese elite malting barley cultivar ‘Haruna Nijo’ and the barley reference genome cultivar ‘Morex’. The screen identified four mutants that developed compound SGs in endosperm. Three of them (*hvisa1-3*, *hvisa1-4*, *hvisa1-5*) had genetic lesions in *HvISA1*, and the other (*hvflo6-2*) had a mutation in *HvFLO6*. We generated a *hvflo6-2 hvisa1-3* double mutant and showed that it had significantly reduced starch biosynthesis and shrunken grains. In contrast to starch, phytoglycogen and sugars accumulated to higher levels in the double mutant than in the single mutants. The results provide new insights into the genetic interactions among starch-related mutations and characterize *hvflo6* mutations as enhancers of the *hvisa1* phenotype in barley.

## Materials and methods

### Plant material and growth conditions

Barley cultivars 'Morex' and 'Haruna Nijo' were provided from NBRP-Barley (http://earth.nig.ac.jp/~dclust/cgi-bin/index.cgi?lang=en). *Risø17* (NGB11438) was obtained from the Nordic Genetic Resource Center (https://www.nordic-baltic-genebanks.org/gringlobal/search.aspx). The *Franubet* seed was provided Dr. Toshiki Nakamura at NARO Tohoku Agricultural Research Center (Saito et al. 2018). Barley was grown at 22 °C/18 °C in a growth cabinet (NK Systems, LPH-411S) or around 23 °C at 16-h day/8-h night conditions. Biomass was measured at 25 days after germination. Transgenic plants were grown at 15 °C/13 °C (16-h day/8-h night) in a growth chamber. Barley *TP-GFP* plants were constructed as described previously (Matsushima and Hisano 2019). Mutagenization was done by imbibing 10,000 grains of Haruna Nijo and 1100 grains of Morex for 20 h in iced water with aeration, then exposed them to 1 mM sodium azide in 100 mM phosphate buffer (pH 3.0) at 20 °C for 3 h with aeration. Following the treatment, the grains were rinsed in running water and then immediately planted at an experimental field at the Institute of Plant Science and Resources, Okayama University, under natural conditions. M2 grains were harvested as a mixture of M1 plants after self-fertilization. A total of 10,000 M2 plants were grown and M3 grains were also harvested from individual M2 plants. The screening was carried out with at least five grains from each M2 and M3 line using the rapid observation method of SGs (Matsushima et al. 2010).

### Observation of SGs by thin-section microscopy with Technovit 7100 Resin

Preparation of Technovit sections to observe SGs in endosperm are described previously (Matsushima et al. 2014). Thin sections (1 μm) were prepared using an ultramicrotome (Leica Microsystems, LEICA EM UC7). To stain SGs, thin sections were stained with 40-times diluted Lugol solution (iodine/ potassium iodine solution; MP Biomedicals, #155,269) in deionized water and subsequently examined with a microscope (Olympus, BX53).

### Preparation of antisera against HvISA1

To obtain antisera against HvISA1, recombinant protein was produced in *E. coli*. First, total RNA was isolated from developing barley grains using a NucleoSpin RNA Plant and Fungi kit (Macherey–Nagel, U0120B). The RNA was used to synthesize cDNA libraries using PrimeScript™ II 1st strand cDNA Synthesis kit (Takara, 6210A, Japan). *HvISA1* cDNA was amplified using the following primers; 5′-GTTACTTCTGCAGGGATGAAGATGATGGCCATGG-3′.and 5′-CCGGGGGATCGGATCTCAAACATCAGGGCGTGAT-3′. The fragment was cloned into a cloning plasmid. Next, the cDNA fragment encoding C-terminal 419 amino acids of HvISA1 without stop codon was amplified using the following primers; 5′-TTTTCATATGCTTGCACCCAAGGGAGAG-3′ and 5′-TTTTCTCGAGAACATCAGGGCGTGATACAAGG-3′. The fragment was cloned into the *Nde*I and *Xho*I sites of pET24a( +) vector (Sigma-Aldrich). Overexpression of the recombinant protein was induced in Shuffle T7 Express Competent *E. coli* cells (New England Biolabs) by adding isopropyl-β-D-thiogalactopyranoside (IPTG, final concentrations of 0.5 mM). After induction, *E. coli* cells were grown at 20 °C for 18 h and then harvested by centrifugation. The recombinant protein was recovered as inclusion bodies. Isolation of inclusion bodies followed by Okegawa et al (2016). *E. coli* cells derived from 100 mL culture were suspended in 10 mL lysis buffer containing 20 mM Tris–HCl (pH 7.6), 500 mM NaCl, and 0.5 mM EDTA, and disrupted by sonication. After centrifugation at 11,000 × g for 10 min, the precipitate was resuspended in 10 mL lysis buffer and centrifuged at 11,000 × g for 10 min. The precipitate was resuspended in a washing buffer containing 20 mM Tris–HCl (pH 7.6), 500 mM NaCl and 4% (w/v) Triton X-100 by sonication and then gently mixed using a rotator for 30 min at room temperature. The inclusion bodies were recovered as precipitate after centrifugation at 11,000 × g for 10 min. The washing step was repeated three times. Finally, the inclusion bodies were washed twice with 10 mL of ice-cold water to thoroughly remove detergent soluble contaminated proteins. The HvISA1 inclusion bodies were subjected to SDS-PAGE. After electrophoresis, the edge of the gel was cut off and stained with 0.1% Coomassie blue R-250 (Cosmo Bio, Japan) and used as a reference to know the position of the recombinant HvISA1 protein in the gel. According to the position, the gel slice containing HvISA1 protein was cut off, and electro-eluted from the gel slices using Model 422 Electro-Eluter (BioRad). After the elution, recombinant HvISA1 protein was concentrated using centrifugal concentrator Vivaspin Turbo 15 (Sartorius). Anti-HvISA1 antisera were raised in rabbits against the purified recombinant protein.

### Western blotting

The developing grains at 15 days after awn emergence (DAA) were cultivated and stored at − 80 °C. The grain was mixed in 500 μL of urea-SDS-sample buffer: 8 M Urea, 5% (v/v) 2-mercaptoehtanol, 4% (w/v) SDS, 125 mM Tris–HCl (pH 6.8), 10 μL mL^−1^ protease inhibitor cocktail (Sigma-Aldrich, P9599) and then crushed using Multi-beads Shocker MB2000 (YASUI KIKAI, Japan). The samples were incubated for 1 h at room temperature and subjected to centrifugation at 16,000 × *g* at 20 °C for 10 min. Supernatants were subjected to SDS-PAGE, and proteins were transferred electrophoretically to a polyvinylidene difluoride membrane (Millipore). The membrane was then incubated in Tris-buffered saline (pH 7.5) plus 0.05% (v/v) Tween-20 with the anti-HvISA1 antisera for 1 h. Dilutions of the antisera were 1:1,000 (v/v). Anti-Rabbit IgG, HRP-Linked Whole Ab Donkey (Cytiva, NA934V) was diluted (1:2,000) as second antibodies. The immunoreactive bands were detected with Immobilon Crescendo Western HRP substrate (Millipore, WBLUR0500).

### Iodine staining of cross sections of developing grain

Cross sections of grains at 20 DAA were immersed in 3% (v/v) glutaraldehyde at 4 °C for 9 h. The sections were washed with water three times and then stained with 50-times diluted Lugol solution. After washing with water, the stained cross sections were observed using the digital microscope RM-2000 (Hirox, Japan). As another method, cross sections were directly stained with 40-times diluted Lugol solution on the glass slide and observed immediately.

### Starch quantification

The total amount of starch in the grain was measured by enzymatic methods using the enzymes in Resistant Starch kit (Megazyme, K-RSTAR). A grain was ground in 500 μL of 80%(v/v) ethanol using the Multi-beads Shocker MB2000. The sample was incubated at 85 °C for 5 min. After the centrifugation at 1800 × *g*, the pellet was resuspended in 1 mL of 80% (v/v) ethanol and centrifuged to obtain a pellet. The pellet was resuspended in 1 mL of 100 mM sodium maleate buffer (pH 6.0) and centrifuged at 1800 × *g*. After washing three times, the pellet was incubated in 1 mL of 100 mM sodium maleate buffer (pH 6.0) containing 5 mg mL^−1^ pancreatic α-amylase and 1.5 unit mL^−1^ amyloglucosidase at 37 °C for 16 h. The supernatant was recovered after centrifugation at 1800 × *g* for 5 min. The pellet was resuspended with 1 mL of 50% (v/v) ethanol and centrifuged again. The supernatant was recovered and put together with the previous supernatant. This procedure was repeated three times. Finally, the supernatant was adjusted to 10 mL with water. The supernatant (110 μL) was mixed with 900 μL of 100 mM sodium acetate buffer (pH 4.5). The 110 μL of the mix was treated with amyloglucosidase at 50 °C for 20 min. The glucose amount in the sample was measured using GOPOD reagent (Megazyme).

### GFP observation in TP-GFP endosperm

GFP-fluorescent amyloplasts in the endosperm of *TP-GFP* were observed as described previously (Matsushima and Hisano 2019).

### Observation of SGs in pollen grain

To stain SGs in mature pollen, anthers at 5 DAA were disrupted with forceps in the 120-times diluted Lugol solution on a glass cover slide. The released pollen were then squashed by putting gentle pressure on a coverslip to release SGs from pollen vegetative cells. The released SGs were observed with a microscope. To observe GFP-fluorescent amyloplasts in pollen, anthers at 4 DAA were disrupted with forceps in water on a glass slide, and released pollen were examined with the laser-scanning confocal microscope (Olympus, FV1000).

### Soluble α-glucan quantification

The total amount of soluble α-glucan in the grain was measured by enzymatic methods using the enzymes in Resistant Starch kit. The weight of two grains was measured and ground in 1400 μL methanol using the Multi-beads shocker MB2000. The sample was incubated at 85 °C for 10 min. After the centrifugation at 1800 × *g* for 10 min, the pellet was resuspended in 1 mL 90% (v/v) methanol and centrifuged again. Washing with 90% methanol was repeated twice to remove methanol-soluble glucans. After drying, the pellet was resuspended in 1 mL water and rotated at room temperature for 20 min. The supernatant was recovered after centrifugation at 1800 × *g* for 20 min. This procedure was repeated four times, and all the supernatant was put together. The supernatant was adjusted to 4 mL with water. The aliquot (250 μL) was mixed with 750 μL of methanol and placed at − 20 °C for 16 h. After centrifugation at 10,000 × g for 5 min, the pellet was incubated in 220 L of 100 mM sodium maleate buffer (pH 6.0) containing 5 mg mL^−1^ pancreatic α-amylase and 1.5 unit mL^−1^ amyloglucosidase at 37 °C for 16 h. The supernatant (110 μL) was mixed with 900 μL of 100 mM sodium acetate buffer (pH4.5). The 110 μL of the mix was treated with amyloglucosidase at 50 °C for 20 min. The glucose amount in the sample was measured using the GOPOD reagent (Megazyme).

### β-glucan quantification

The total amount of β-glucan in the grain was measured using the enzymes in Mixed-linkage beta-glucan kit (Megazyme, K-BGLU). A grain was ground in 1 mL 50% (v/v) ethanol using Multi-beads shocker MB2000. The sample was incubated at 100 °C for 5 min. After the centrifugation at 1800 × *g* for 10 min, the pellet was resuspended in 1 mL 50% (v/v) ethanol and centrifuged at 2800 × *g* for 10 min. The pellet was resuspended in 1 mL of 20 mM sodium phosphate buffer (pH 6.5) and mixed with 2.5 units of lichenase. The sample was incubated at 50 °C for 1 h. Then, the sample was adjusted to 4 mL with 200 mM sodium acetate buffer (pH 4.5). The aliquot (50 μL) was mixed with β-glucosidase at 50 °C for 10 min. The glucose amount in the sample was measured using GOPOD reagent in the kit.

### Glucose chain length distribution of total α-glucan in grain

The glucose chain-length distribution of total α-glucan was analyzed using fluorescence labeling and capillary electrophoresis (Beckman Coulter). The procedures were the same as in our previous study (Matsushima et al. 2010).

### Quantification of sugars by gas chromatography-mass spectrometry (GC–MS)

Extraction and quantification of sugars were followed as described by Raviv et al. (2020). Briefly, two grains were ground using Multi-beads Shocker MB2000. The samples were extracted in 1.4 mL of 100% methanol with ribitol (12 μg) supplemented as an internal standard. The samples were homogenized and incubated at 70 °C with shaking for 10 min. The samples were centrifuged for 10 min at 12,000 × *g*. The supernatant was taken and vigorously mixed with 0.7 mL chloroform and 1.5 mL ice-cold water. The phases were separated by centrifugation at 2000 × *g* for 15 min, and 150 μL of the upper polar phase was sampled and dried in a vacuum concentrator. The dried samples were sequentially derivatized with methoxyamine hydrochloride and* N*-methyl-*N*-trimethylsilyl-trifluoroacetamide (Tokyo Chemical Industry). The derivatized samples were diluted by ten times with dichloromethane (FUJIFILM, Japan), and metabolites were analyzed with an Agilent 7890A-GC/240-MS instrument (Agilent Technologies). Mass spectra data were collected in full scan mode in mass range *m/z* 40–750. Metabolites were identified by comparing their fragmentation patterns with Mass Spectral Library (National Institute of Standards and Technology).

### Reverse transcription-PCR

Total RNA was extracted from developing grains at 15 DAA using NucleoSpin RNA Plant and Fungi kit. Two hundred fifty nanograms of total RNA were used for first-stranded cDNA synthesis using PrimeScript™ II 1st strand cDNA Synthesis kit and oligo dT primers. The PCR conditions were as follows: 94 °C for 2 min and 30–35 cycles of 94 °C for 30 s, 53 °C for 45 s, and 68 °C for 1 min. The primers used were as follows: *HvFLO6* gene, 5′-GGTCTTTTGATGGATGGACAAG-3′ and 5′- CTTCCAAACACCATCAACAATG-3′; *ACTIN* gene, 5′-AAGTACAGTGTCTGGATTGGAGGG-3′ and 5′- TCGCAACTTAGAAGCACTTCCG-3′. PCR products were subsequently separated by 15% PAGE and detected with ethidium bromide staining.

### Genotyping of hvisa1-3 and hvflo6-2 mutations

The base change of *hvisa1-3* mutation was detected by the derived cleaved-amplified polymorphic sequence primers: 5′-CTTGAAAGTCCCTCTTATTCCTTTC-3′ and 5′-CTTACCTTCCCATTCCACTCAGACCAAAGATT-3′. The PCR conditions were as follows: 94 °C for 2 min and 35 cycles of 94 °C for 30 s, 53 °C for 45 s, and 68 °C for 1 min. The PCR product was digested with *Hinf*I, and PCR products were subsequently separated by 15% PAGE and detected with ethidium bromide staining. In the case of wild type, a PCR product (134 bp) was digested into 102 and 32 bp. In the case of *hvisa1-3*, the PCR product was not digested.

To detect the base changes of *hvflo6-2* mutation, PCR products were amplified with two different sets of primers, wild type-specific primer sets (5′-CACCAGGCTGCGGAGAGAGAGGG-3′ and 5′-CCTTCACAGGCAGTAACTAAACAGT-3′) and *hvflo6-2* specific primer sets (5′-CACCAGGCTGCGGAGAGAGAGTT-3′ and 5′-CCTTCACAGGCAGTAACTAAACAGT-3′). The PCR conditions were as follows: 94 °C for 2 min and 35 cycles of 94 °C for 30 s, 58 °C for 45 s, and 68 °C for 1 min. PCR products were subsequently separated by 15% PAGE and detected with ethidium bromide staining. The wild-type specific primer sets can amplify 266-bp PCR product from wild type allele but not from *hvflo6-2* allele. In contrast, *hvflo6-2* specific primer sets can amplify 266-bp PCR products only from *hvflo6-2* allele but not from wild type allele. *hvflo6-1* mutation site was detected according to the previous paper (Saito et al. 2018).

### ADP-glucose quantification

ADP-glucose quantification was followed by Park et al. (2010). Approximately 60 mg of grains at 15 DAA were homogenized to a fine powder under liquid nitrogen, and 15 mM tetrabutylammonium hydrogen sulfate (pH 1.9) was added to the homogenate. After 15 min stirring on ice, samples were centrifuged at 10,000 × *g* for 10 min, and supernatants were filtered at 12,000 × *g* for 30 min through Amicon Ultra centrifugal filters with 3 kDa molecular weight cutoff (Merck). The flow-through fractions were applied to DEAE-5PW column (7.5 × 75 mm, Tosoh) at a flow rate of 1 ml min^−1^. Two eluents, A [10 mM ammonium acetate buffer (pH 8.0)] and B [800 mM ammonium acetate buffer (pH 4.8)], were used. The column was equilibrated with eluent A. After injecting a sample, the column was eluted with a linear gradient of eluent B to 50% in 15 min, to 100% in 5 min, and was eluted with eluent B to last 10 min to obtain the fraction containing ADP-glucose. The fraction was further analyzed by an Inertsil ODS-3 column (GL Sciences, Japan) at a flow rate of 1.5 ml min^−1^ using isocratic elution with 50 mM triethylamine phosphate buffer (pH 6.5). Commercial ADP-glucose (Cayman Chemical) was used as the standard to quantify the concentration. ADP-glucose was monitored by the absorbance at 262 nm.

### The water content of grains

The grains were stored in a desiccator at 10% relative humidity and room temperature for more than one month. Grains were weighed and dried at 150 °C for 3 h, then reweighed. Water contents were calculated from these data.

### Native-PAGE activity staining of SS, BE, DBE and PULLULANASE (PUL)

Soluble proteins were extracted from developing barley grains at 13–15 DAA using 2 volumes (3 volumes for BE) of grinding solution [50 mM imidazole–HCl (pH 7.4), 8 mM MgCl_2_, 500 mM 2-mercaptoethanol, 0.1% (w/v) acarbose, 12.5% (v/v) glycerol] relative to grain fresh weight. Developing grains (10–15 DAA) of rice (*Oryza sativa* subspecies *japonica* “Nipponbare”) grown in a paddy field of Akita Prefectural University during the summer months under natural condition was used as control. SS activity staining was performed using a gel containing 0.8% (w/v) oyster glycogen (Sigma-Aldrich, G8751) as primer (Miura et al. 2018). BE activity staining was assessed using a gel containing 0.0001% oyster glycogen (Yamanouchi and Nakamura 1992). DBE activity staining was performed using gels containing 0.4% (w/v) potato amylopectin (Sigma-Aldrich, A8515) as described in Fujita et al. (1999). PUL activity staining was performed using a gel including 1.34% (w/v) red pullulan (Megazyme) as described in Fujita et al. (2009).

## Results

### Isolation of barley mutants with altered SG morphology

**Fig. 1:**
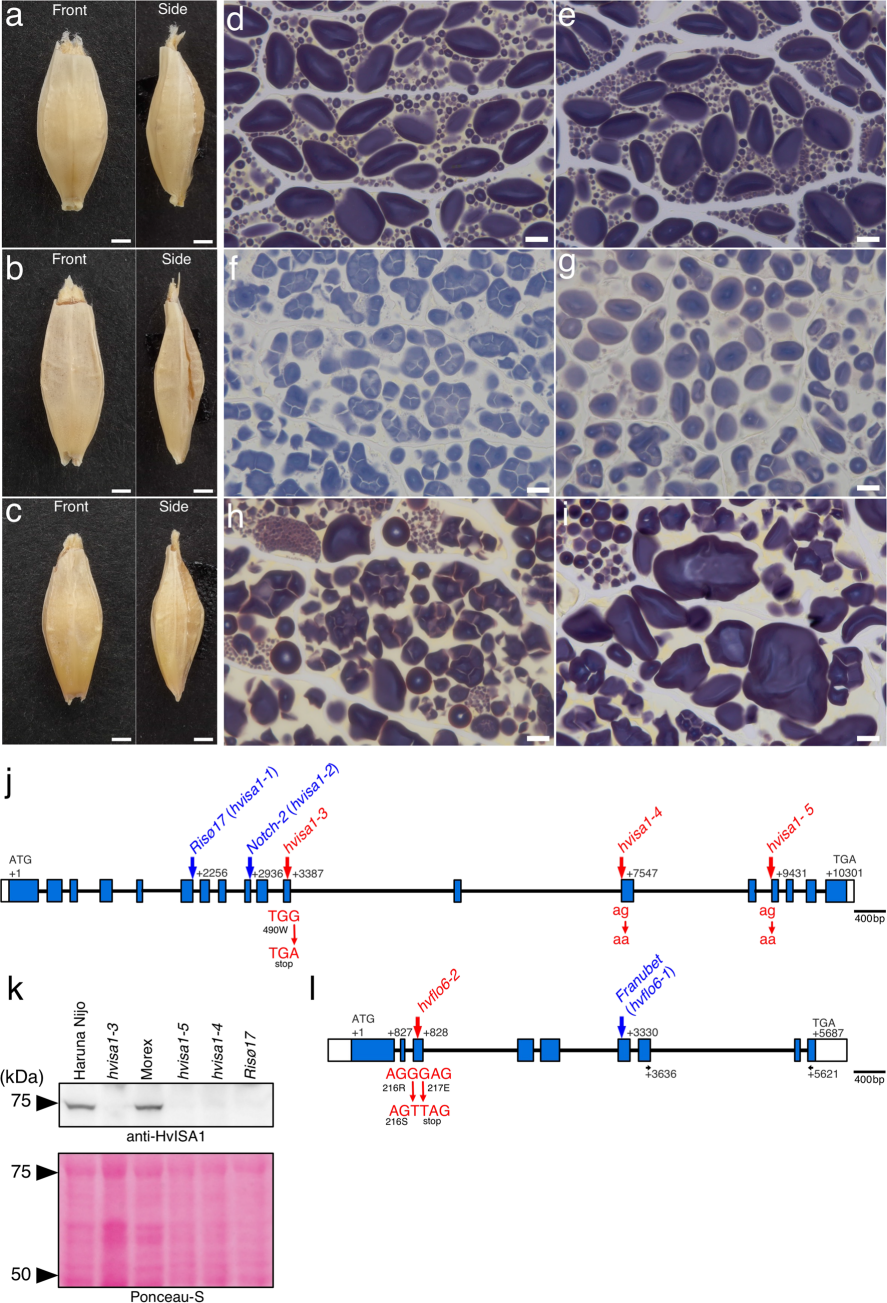
Isolation of new mutant alleles of *HvISA1* and *HvFLO6* in barley. **a**–**c** Mature grains of Haruna Nijo (a) and the new mutants *hvisa1-3* (b) and *hvflo6-2* (c). Front and side views are shown. Bars = 1 mm. **d–i** Iodine-stained thin sections of endosperm cells of Haruna Nijo (d), Morex (e), *hvisa1-3* (f and g), and *hvflo6-2* (h and i). Bars = 10 μm. **j** Structure of the *HvISA1* gene on chr7H: 185,468,510..185,479,005 in MorexV3. The coding and untranslated regions are depicted as blue and white boxes, respectively. Introns are indicated by black lines. The exon–intron structure is based on the reported full-length cDNA (AB074189). The adenine in the translation start codon (ATG) is designated as + 1. *hvisa1-3* has a base pair change from G to A at + 3387. This change introduces a TGA termination codon at the position of tryptophan (W)-490. The mutations in *hvisa1-4* and *hvisa1-5* are located at the splicing acceptor sites of the 12th and 14th introns, respectively. The reported sites of the *Risø17* and *Notch-2* mutations are indicated (Burton et al. 2002). **k** Immunoblot analysis using anti-HvISA1 antisera. The Ponceau S-stained membrane is shown. **l** Structure of the *HvFLO6* gene on chr4H: 9,807,808..9,814,176 in MorexV3. The exon–intron structure is based on the reported full-length cDNA (AK373583). *hvflo6-2* has two consecutive base pair changes at + 827 and + 828. The former change results in an amino acid substitution from arginine (R) to serine (S) at position 216. The latter change introduces a premature stop codon at glutamate (E)-217. The previously reported site of the *Franubet* (*hvflo6-1*) mutation is also indicated (Saito et al. 2018). The primer positions that were used for RT-PCR are indicated by black arrows

To isolate barley mutants with altered SG morphology, we observed SGs in the endosperm of barley grains from a mutagenized population using our method for rapid SG observation that is based on the hand sectioning developed previously (Matsushima et al. 2010). We screened 194 M2 lines and 4325 M3 lines from a Haruna Nijo mutagenized population and 226 M2 lines from a Morex mutagenized population. At least five grains were screened for each line. From the Haruna Nijo mutagenized population, we isolated *hvisa1-3* and *hvflo6-2* mutants, which develop compound SGs in endosperm. Mature grains of the mutants were slightly smaller than those of the wild type (Fig. 1a–c). Iodine-stained Technovit thin sections of the endosperm showed the morphologies of the SGs. The wild-type barley cultivars Haruna Nijo and Morex developed typical bimodal SGs in the endosperm (Fig. 1d, e). In *hvisa1-3* endosperm, some cells developed compound SGs, and other cells developed simple SGs (Fig. 1f–g). We also observed cells in which both compound and simple SGs coexist. In the *hvflo6-2* mutant, some cells developed compound SGs, and other cells developed enlarged simple SGs (Fig. 1h, i).

A previous study showed that the barley *Risø17* and *Notch-2* mutants have lesions in the *HvISA1* gene and develop compound SGs in endosperm cells (Burton et al. 2002). The phenotypic similarities between *Risø17*, *Notch-2*, and *hvisa1-3* encouraged us to determine the sequence of the *HvISA1* gene in *hvisa1-3*. We amplified the genomic region (chr7H: 185,468,510.185,479,005 in MorexV3) covering the *HvISA1* cDNA sequence (AB074189) from *hvisa1-3* and determined the sequence. In *hvisa1-3*, the guanine residue located 3387 bp downstream of the first ATG was replaced by adenine (Fig. 1j). The mutation introduced a premature stop codon at amino acid position 490. We also isolated two additional mutants (*hvisa1-4* and *hvisa1-5*) with similar phenotypes to *hvisa1-3* from the Morex mutagenized population. The *hvisa1-4* and *hvisa1-5* mutants had base changes from guanine to adenine at 7547 bp and 9431 bp downstream of the first ATG, respectively (Fig. 1j). Both of these mutations were located at putative splicing acceptor sites. *Risø17*, *hvisa1-4*, and *hvisa1-5* developed compound SGs in the endosperm (Supplementary Fig. 1a–c). Immunoblot analysis showed that *hvisa1-3*, *hvisa1-4*, *hvisa1-5*, and *Risø17* lacked HvISA1 protein in developing grains, while the wild-type parental cultivars Haruna Nijo and Morex accumulated HvISA1 (Fig. 1k). These results indicate that not only the premature stop codon but also impaired splicing of *HvISA1* transcripts prevented the accumulation of HvISA1 protein. From these results, we concluded that *hvisa1-3*, *hvisa1-4*, and *hvisa1-5* are allelic to *Risø17* and *Notch-2.* In this study, we renamed *Risø17* and *Notch-2* as *hvisa1-1* and *hvisa1-2*, respectively (Fig. 1j).

The *Franubet* mutant developed enlarged simple SGs and compound SGs in the endosperm (Supplementary Fig. 1d, e), which resembled the SGs in *hvflo6-2*. To identify the specific base change in the *HvFLO6* gene of *hvflo6-2*, we amplified the genomic region (chr4H: 9,807,808.9,814,176 in MorexV3) covering the *HvFLO6* cDNA sequence (AK373583) and determined the sequence. In *hvflo6-2*, two consecutive guanine residues at + 827 and + 828 were changed to adenines (Fig. 1l). The latter change introduced a premature stop codon at aa position 217. RT-PCR showed that the *HvFLO6* gene was expressed in the *hvflo6-2* mutant but at a lower level than in the wild type (Supplementary Fig. 2). The F1 grain from a cross between *hvflo6-2* and *Franubet* developed compound SGs and enlarged simple SGs, just like the parents (Supplementary Fig. 1f, g). In addition, all F2 progeny (n = 36) of the cross between *hvflo6-2* and *Franubet* showed mutant phenotypes. These results indicate that *hvflo6-2* is allelic to *Franubet*. Hereafter, therefore, we refer to the *Franubet* mutant as *hvflo6-1.*

We designed derived cleaved-amplified polymorphic sequence (dCAPS) primers to detect the base change in *hvisa1-3*. The dCAPS primers successfully genotyped Haruna Nijo, heterozygous *hvisa1-3*^+*/–*^, and homozygous mutation of *hvisa1-3* (Supplementary Fig. 3a). We also designed two sets of primer pairs to detect the base changes in *hvflo6-2*. These primer sets successfully genotyped Haruna Nijo, heterozygous *hvflo6-2*^+*/–*^, and homozygous mutation of *hvflo6-2* (Supplementary Fig. 3b).

### Construction of the hvflo6-2 hvisa1-3 double mutant

Next, we generated the *hvflo6-2 hvisa1-3* double mutant to investigate the effect of genetic interaction between these alleles on SG morphology*.* Both *hvisa1-3* and *hvflo6-2* are in the Haruna Nijo background, so the *hvflo6-2 hvisa1-3* double mutant is also in the Haruna Nijo background, thus avoiding confounding effects of genetic background. Like *hvisa1-3*, the *hvflo6-2 hvisa1-3* double mutant did not accumulate HvISA1 protein (Supplementary Fig. 4). Plant appearance at 25 days after germination showed that the growth of *hvflo6-2* and *hvflo6-2 hvisa1-3* mutants was impaired compared to Haruna Nijo and *hvisa1-3* (Supplementary Fig. 5a–d). The *hvflo6-2* plants had significantly fewer tiller numbers and reduced shoot fresh weight compared to Haruna Nijo and the other mutants (Supplementary Fig. 5e, f). The *hvisa1-3* plants showed a slight but not significant growth impairment compared to Haruna Nijo. The growth of *hvflo6-2 hvisa1-3* was also inhibited compared to Haruna Nijo, but to a lesser extent than the *hvflo6-2* plants*.* The growth defect of *hvflo6-2 hvisa1-3* was presumably due to the *hvflo6-2* mutation. Growth impairment was also reported for *hvflo6-1* (Watson-Lazowski et al. 2022). When grains were assessed for germination on moistened filter paper, germination ratio of *hvisa1-3* (95.0%, *n* = 40), *hvflo6-2* (95.2%, *n* = 42) and *hvflo6-2 hvisa1-3* grains (97.9%, *n* = 48) was almost the same as that of Haruna Nijo (98.0%, *n* = 51).

**Fig. 2:**
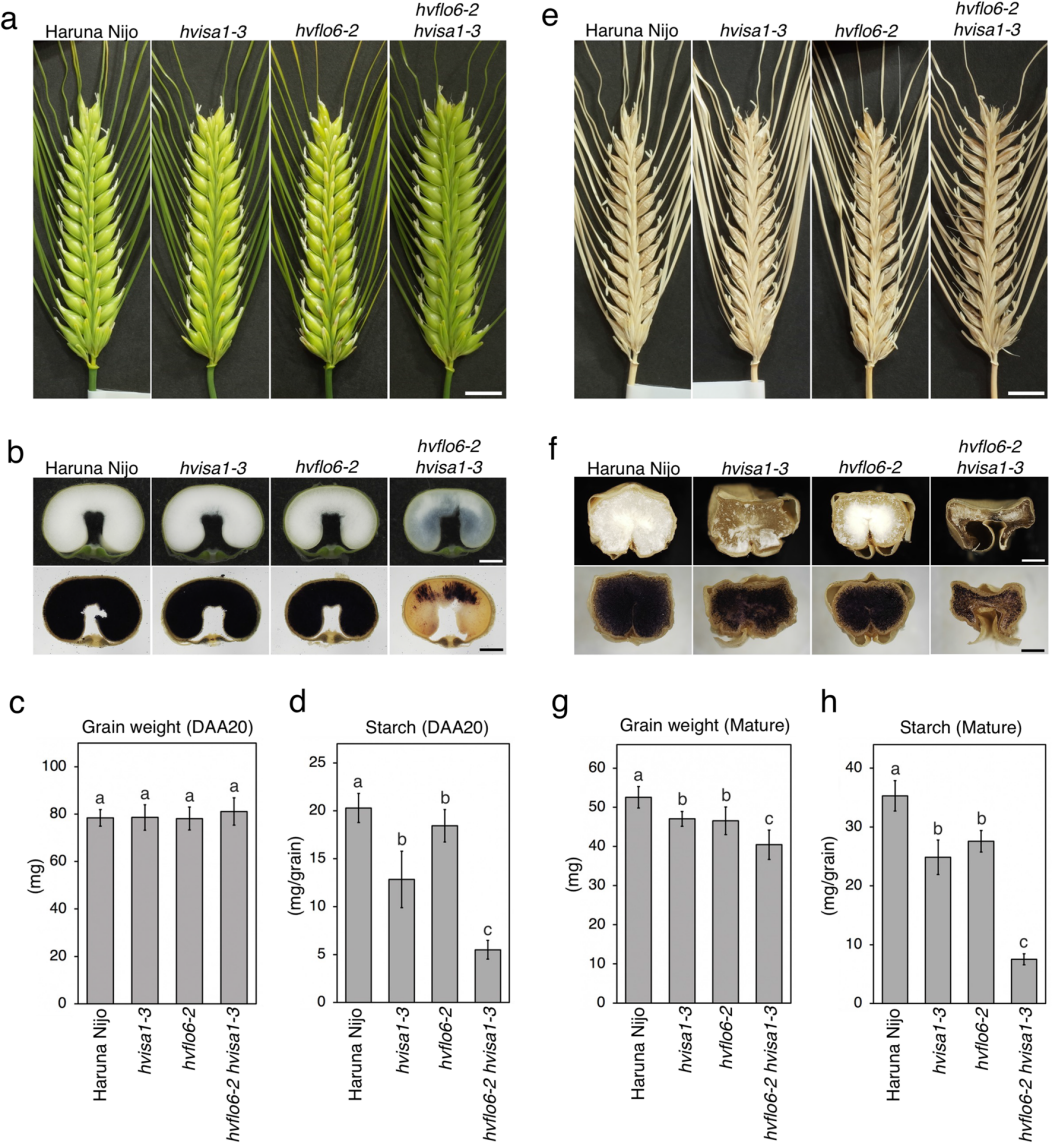
Generation of the *hvflo6-2 hvisa1-3* double mutant. **a** Panicles of Haruna Nijo, *hvisa1-3*, *hvflo6-2*, and *hvflo6-2 hvisa1-3* at 20 days after awn emergence (DAA). Bar = 1 cm. **b** Unstained (top) and iodine-stained (bottom) cross sections of grains at 20 DAA. Bars = 1 mm. **c** Single grain weight at 20 DAA (*n* = 4). **d** Starch amount per grain at 20 DAA (*n* = 4). **e** Mature panicles of Haruna Nijo, *hvisa1-3*, *hvflo6-2*, and *hvflo6-2 hisa1-3*. Bar = 1 cm. **f** Unstained (top) and iodine-stained (bottom) cross sections of mature grains. Bars = 1 mm. **g** Single grain weight at the mature stage (*n* = 6). **h** Starch amount per grain at the mature stage (*n* = 3). (c, d, g, h) Data are given as means ± SD. Statistical comparisons were performed using Tukey’s HSD. The same letters above the bars represent statistically indistinguishable groups, and different letters represent statistically different groups (*p* < 0.05)

The appearance of *hvflo6-2 hvisa1-3* panicles was similar to that of Haruna Nijo and the single mutants at 20 days after awn emergence (DAA) (Fig. 2a). Endosperm cross sections showed that the translucent area was appeared in *hvflo6-2 hvisa1-3* compared to Haruna Nijo, while endosperm cross-sections of single mutants were indistinguishable with Haruna Nijo (Fig. 2b, upper row). Iodine staining of grain cross sections showed that starch accumulation was not uniform in *hvflo6-2 hvisa1-3* (Fig. 2b, lower row). Grain weights at 20 DAA were almost the same for Haruna Nijo and the mutants (Fig. 2c). In contrast, the amount of starch in the grains at 20 DAA was significantly decreased in *hvflo6-2 hvisa1-3* compared to Haruna Nijo and the single mutants (Fig. 2d). At the mature stage, *hvflo6-2 hvisa1-3* grains were shrunken (Fig. 2e, f). The grain weight and starch content of mature *hvflo6-2 hvisa1-3* grains were significantly reduced compared to those of Haruna Nijo and the single mutants (Fig. 2g, h).

To confirm the association between the double mutation and the shrunken grain phenotype, grains of selfed *hvflo6-2*^+*/–*^* hvisa1-3* plants were subjected to co-segregation analysis. *hvflo6-2*^+*/–*^ indicates the heterozygosity of *hvflo6-2* allele. The panicles of *hvflo6-2*^+*/–*^* hvisa1-3* plants produced shrunken grains (Supplementary Fig. 6a): 30 of 112 grains showed a shrunken phenotype, and the Chi-square value for 1:3 segregation was 0.19 (*p* > 0.5). Shrunken grains and normal grains could be easily distinguished by observing the cross sections (Supplementary Fig. 6b). Genotyping of the grains showed that all shrunken grains were associated with the *hvflo6-2 hvisa1-3* double homozygous genotype, whereas normal grains were *hvflo6-2*^+*/–*^* hvisa1-3* or *HvFLO6 hvisa1-3* (Supplementary Fig. 6c). This suggests that the homozygous *hvflo6-2* allele is needed to cause the shrunken phenotype of grains of *hvisa1-3* background. This genetic data confirmed that the *hvflo6-2 hvisa1-3* double mutation impaired starch biosynthesis rather than single mutants and caused a shrunken phenotype.

### Morphology of SGs in hvflo6-2 hvisa1-3 endosperm

**Fig. 3:**
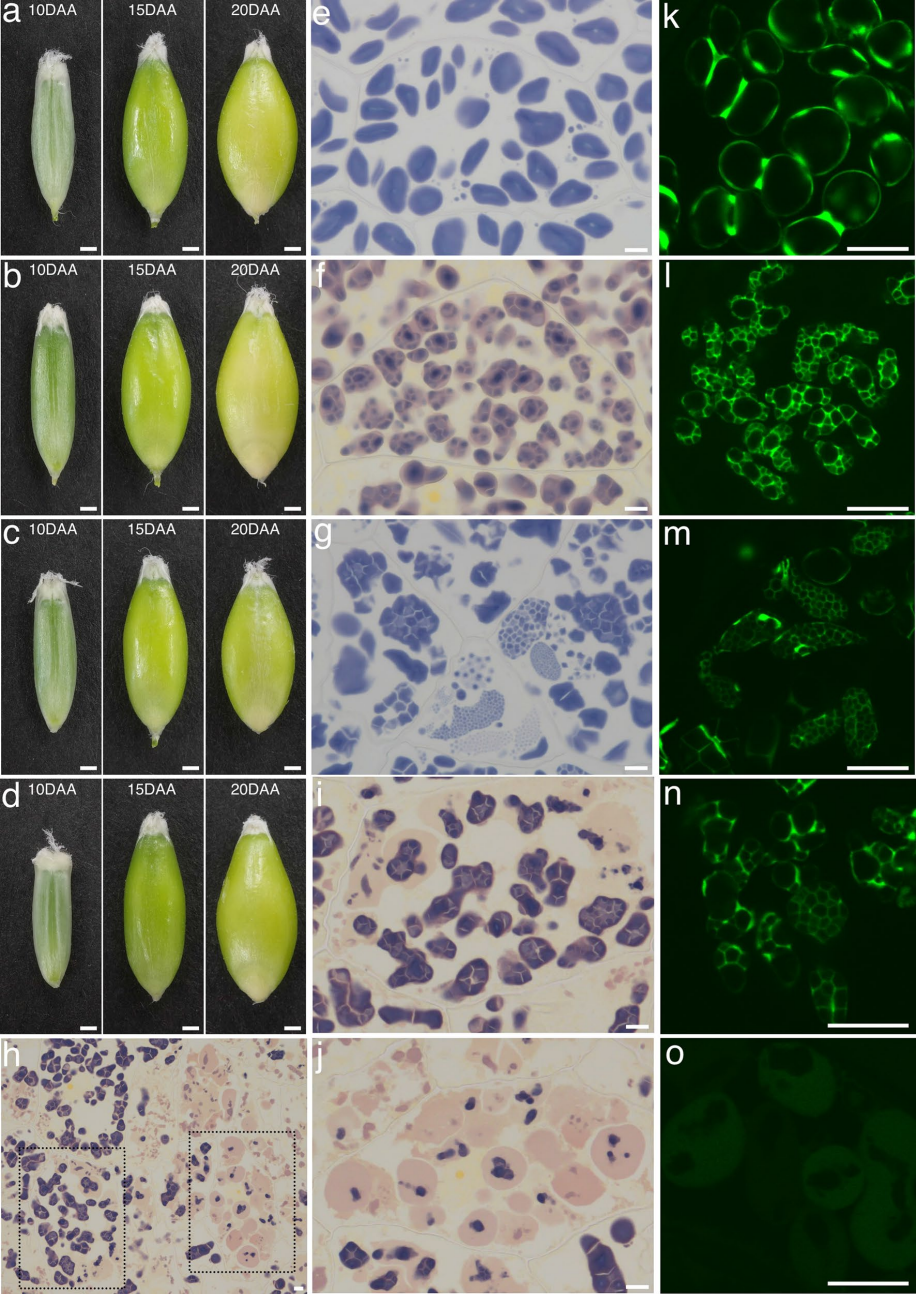
Starch granule (SG) morphology in developing grains. **a–d** Dehulled developing grains of Haruna Nijo (a), *hvisa1-3* (b), *hvflo6-2* (c), and *hvflo6-2 hvisa1-3* (d) at 10 DAA, 15 DAA, and 20 DAA. Bars = 1 mm. **e–j** Iodine-stained thin sections of endosperm cells at 15 DAA of Haruna Nijo (e), *hvisa1-3* (f), *hvflo6-2* (g), and *hvflo6-2 hvisa1-3* (h–j). Compound SGs are visible in the starch-rich regions of *hvflo6-2 hvisa1-3* endosperm (i) and balloon-like pinkish-stained structures are visible in the starch-less regions (j). Purple-stained areas are visible inside the pinkish structures. (i) and (j) are the magnified images of the dotted areas in (h). Bars = 10 μm. **k–o** Fluorescence images of amyloplasts in 15 DAA grains of plants expressing *TP-GFP*. Bar = 10 μm. Amyloplasts of Haruna Nijo (k), *hvisa1-3* (l), *hvflo6-2* (m), and the starch-rich regions of *hvflo6-2 hvisa1-3* (n), as well as the balloon-like structures in the starch-less regions of *hvflo6-2 hvisa1-3* (o). Bars = 10 μm. Image-capturing conditions were identical for all GFP fluorescence images

Next, we investigated the change of the SG morphology in *hvflo6-2 hvisa1-3* grains. First, we examined SGs in developing endosperm at 10 DAA, 15 DAA, and 20 DAA (Fig. 3). At these three stages, all mutants showed similar grain development to Haruna Nijo (Fig. 3a–d). Haruna Nijo produced two sizes of simple SGs during grain development (Fig. 3e), with the larger A-type SGs forming earlier and the smaller B-type SGs forming later (Supplementary Fig. 7a, b). In the *hvisa1-3* and *hvflo6-2* single mutants, compound SGs were observed (Fig. 3f, g; Supplementary Fig. 7c–f). In the *hvflo6-2 hvisa1-3* double mutant, endosperm differentiated into two regions that stained differently with iodine, one purple and the other pinkish (Fig. 3h). Compound SGs were observed in the purple-stained regions (Fig. 3i), while the pinkish regions contained balloon-like structures that mainly stained pinkish but had internal areas stained purple (Fig. 3j). The pinkish region was not detected in the iodine-stained cross sections of *hvflo6-2 hvisa1-3* endosperm (Fig. 2b), perhaps because the pinkish materials were water-soluble and washed out after staining. Therefore, we examined cross sections immediately after iodine staining without washing. Pinkish regions were detected in cross sections of *hvflo6-2 hvisa1-3* endosperm, but not Haruna Nijo or single mutant endosperms (Supplementary Fig. 8a–d).

To confirm the altered SG morphologies in the mutants by a different method, we used a transgenic barley line carrying a transit peptide (TP) linked to green fluorescent protein (GFP); the plastid-targeted TP-GFP localizes to amyloplasts in the endosperm (Matsushima and Hisano 2019). In *TP-GFP* plants, GFP is transported into the stroma of amyloplasts but cannot penetrate into SGs. Therefore, SGs are observed as black spaces in GFP-labeled amyloplasts. Consistent with previous observations, the GFP signal surrounded the SGs (Fig. 3k). The GFP signal was particularly intense between the SGs where the stroma is located (Matsushima and Hisano 2019). We crossed *TP-GFP* plants with the mutants to visualize amyloplasts and SGs in the mutants. In *hvisa1-3* and *hvflo6-2*, the GFP signal surrounded many small SGs (Fig. 3l, m), indicating that more SGs formed inside amyloplasts of *hvisa1-3* and *hvflo6-2* compared to the wild type. In the *hvflo6-2 hvisa1-3* double mutant, the GFP signal also surrounded multiple small SGs (Fig. 3n). In addition, dilated balloon-like structures with faint GFP fluorescence were observed in the double mutant (Fig. 3o). These structures were similar to those that were stained pinkish by iodine (Fig. 3j). The balloon-like structures contained black areas without GFP fluorescence, which probably correspond to the regions that stained purple with iodine (Fig. 3j). We also noticed GFP-labeled amorphous amyloplasts existed in *hvflo6-2* and *hvflo6-2 hvisa1-3* (Supplementary Fig. 9a, b), which the *hvflo6-2* mutation might cause. These amorphous amyloplasts were often observed in different focal planes from the amyloplasts that contained larger SGs in Fig. 3.

The mature endosperm of *hvflo6-2 hvisa1-3* also differentiated into starch-rich and starch-less regions (Supplementary Fig. 10a). The starch-rich region contained compound SGs (Supplementary Fig. 10b), whereas the formation of SGs was severely impaired in the starch-less region (Supplementary Fig. 10c).

To further confirm the effect of loss of function of both *HvFLO6* and *HvISA1* on SG morphology, we generated a double mutant using a different mutant allele of *HvFLO6* by crossing *hvisa1-3* with *hvflo6-1* to produce *hvisa1-3 hvflo6-1*. The phenotype of this double mutant was the same as that of *hvflo6-2 hvisa1-3*: shrunken grains, starch accumulation in only part of the endosperm, differential staining with iodine, and the presence of compound SGs and abnormal balloon-like structures in the amyloplasts (Supplementary Fig. 11). This observation confirmed that these phenotypes were caused by the double mutation of *HvISA1* and *HvFLO6.*

### Increased abundance of compound SGs in hvflo6-2 hvisa1-3 pollen

**Fig. 4:**
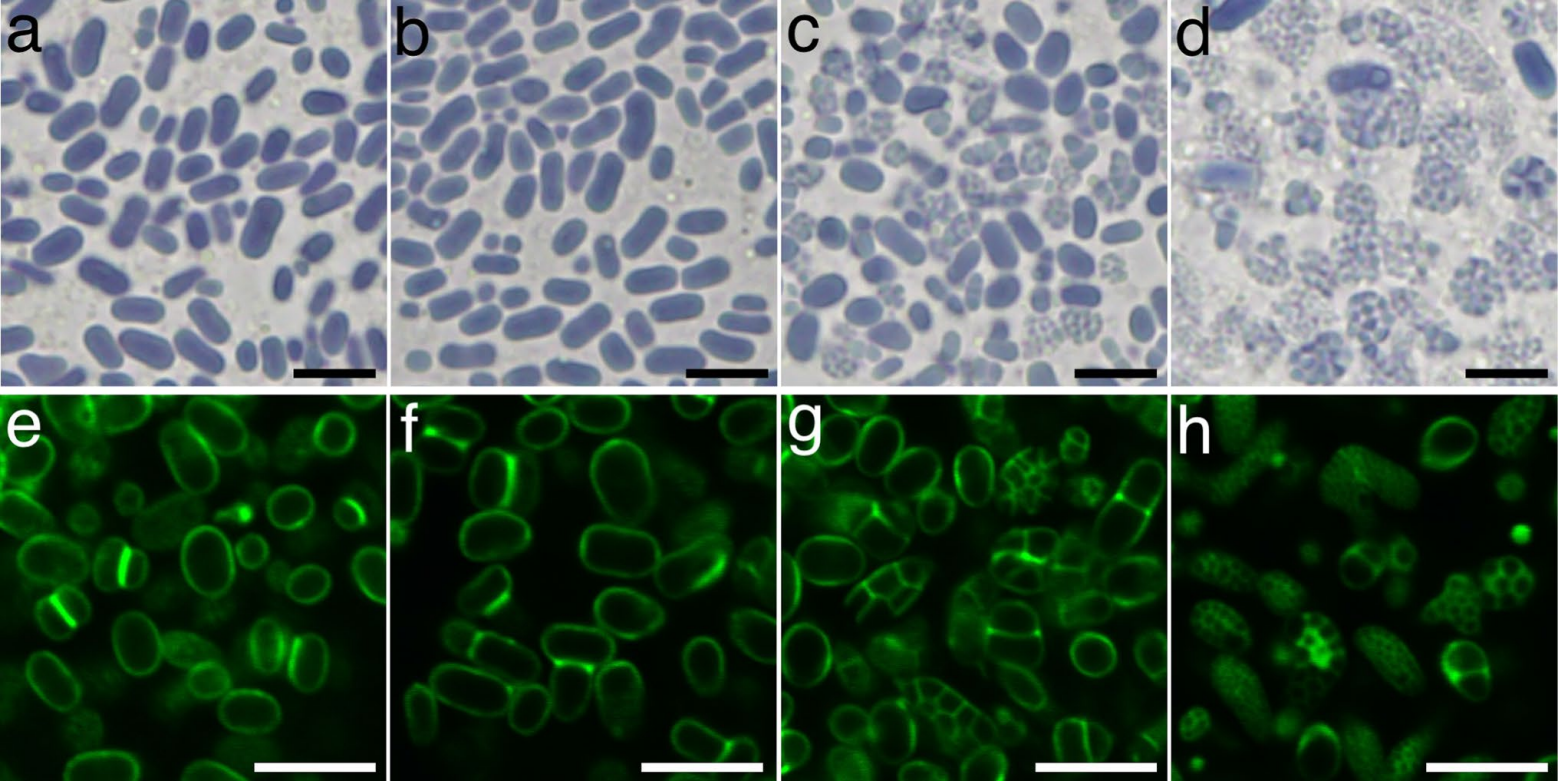
Altered morphologies of SGs and amyloplasts in mutant pollen grains. **a–d** Iodine-stained SGs released from squashed pollen grains of Haruna Nijo (a), *hvisa1-3* (b), *hvflo6-2* (c), and *hvflo6-2 hvisa1-3* (d). Bars = 5 μm. **e**–**h** Fluorescence images of amyloplasts in pollen grains of plants expressing *TP-GFP*: Haruna Nijo (e) *hvisa1-3* (f), *hvflo6-2* (g), and *hvflo6-2 hvisa1-3* (h). Bars = 5 μm

In cereals, starch accumulates in pollen grains as well as the endosperm. When iodine-stained pollen grains from Haruna Nijo and *hvisa1-3* were squashed, rod-shaped SGs were released (Fig. 4a, b). In addition to the rod-shaped SGs, *hvflo6-2* pollen contained SGs formed from smaller starch particles (Fig. 4c), which we designated as compound SGs. The compound SGs were predominant in *hvflo6-2 hvisa1-3* (Fig. 4d). This observation was confirmed using the *TP-GFP* reporter*.* In *hvisa1-3 TP-GFP* pollen, the amyloplast-localized GFP signal surrounded rod-shaped SGs like those observed in wild-type *TP-GFP* pollen (Fig. 4e, f). In contrast, in *hvflo6-2 TP-GFP* pollen, some amyloplasts contained compound SGs (Fig. 4g). In *hvflo6-2 hvisa1-3 TP-GFP* pollen, amyloplasts containing compound SGs became dominant (Fig. 4h). These results indicate that *hvflo6-2* caused the formation of compound SGs in pollen, and this phenotype was enhanced in *hvflo6-2 hvisa1-3.*

### Enhanced phytoglycogen accumulation in hvflo6-2 hvisa1-3 grain

**Fig. 5:**
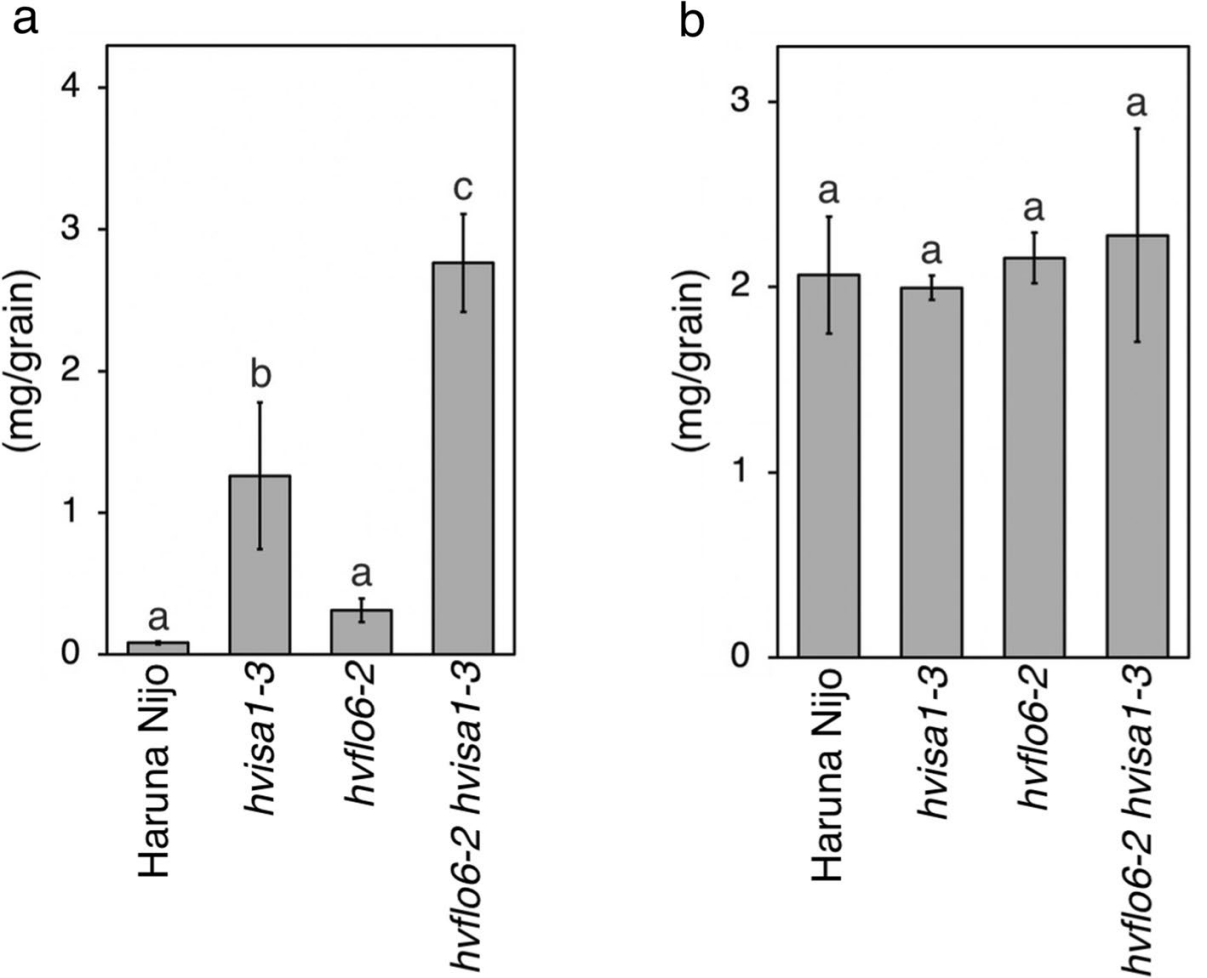
Phytoglycogen accumulation in grains of *hvflo6-2 hvisa1-3*. **a** Amount of soluble α-glucan per mature grain in Haruna Nijo and the single and double mutants (*n* = 4). **b** Amount of β-glucan per mature grain (*n* = 3). Data are given as means ± SD. Statistical comparisons were performed using Tukey’s HSD. The same letters above the bars represent statistically indistinguishable groups, and different letters represent statistically different groups (*p* < 0.05)

Phytoglycogen accumulates in the endosperm of various *isa1* mutants from rice, maize, and barley (Pan and Nelson 1984; Nakamura et al. 1997; Burton et al. 2002). In the rice *isa1* mutant, phytoglycogen-accumulating amyloplasts were stained pinkish by iodine (Nagamatsu et al. 2022), and resembled the balloon-like structures observed in *hvflo6-2 hvisa1-3* and *hvisa1-3 hvflo6-1* (Fig. 3j, Supplementary Fig. 11e). Therefore, we examined the amount of phytoglycogen in the endosperm of the single and double mutants in this study (Fig. 5a). In Haruna Nijo, the phytoglycogen level was low. In contrast, the phytoglycogen level in *hvisa1-3* was more than 15 times higher than that in Haruna Nijo. Phytoglycogen accumulation was slightly higher in *hvflo6-2* than in Haruna Nijo, but the difference was not statistically significant. In *hvflo6-2 hvisa1-3*, phytoglycogen accumulation was almost twice that in *hvisa1-3*. This result means that loss of HvFLO6 enhanced the *hvisa1* mutant phenotype. In contrast, the amount of soluble β-glucan in grains was almost the same in Haruna Nijo and the mutants (Fig. 5b).

### Chain length distribution of total α-glucans in mutants

**Fig. 6:**
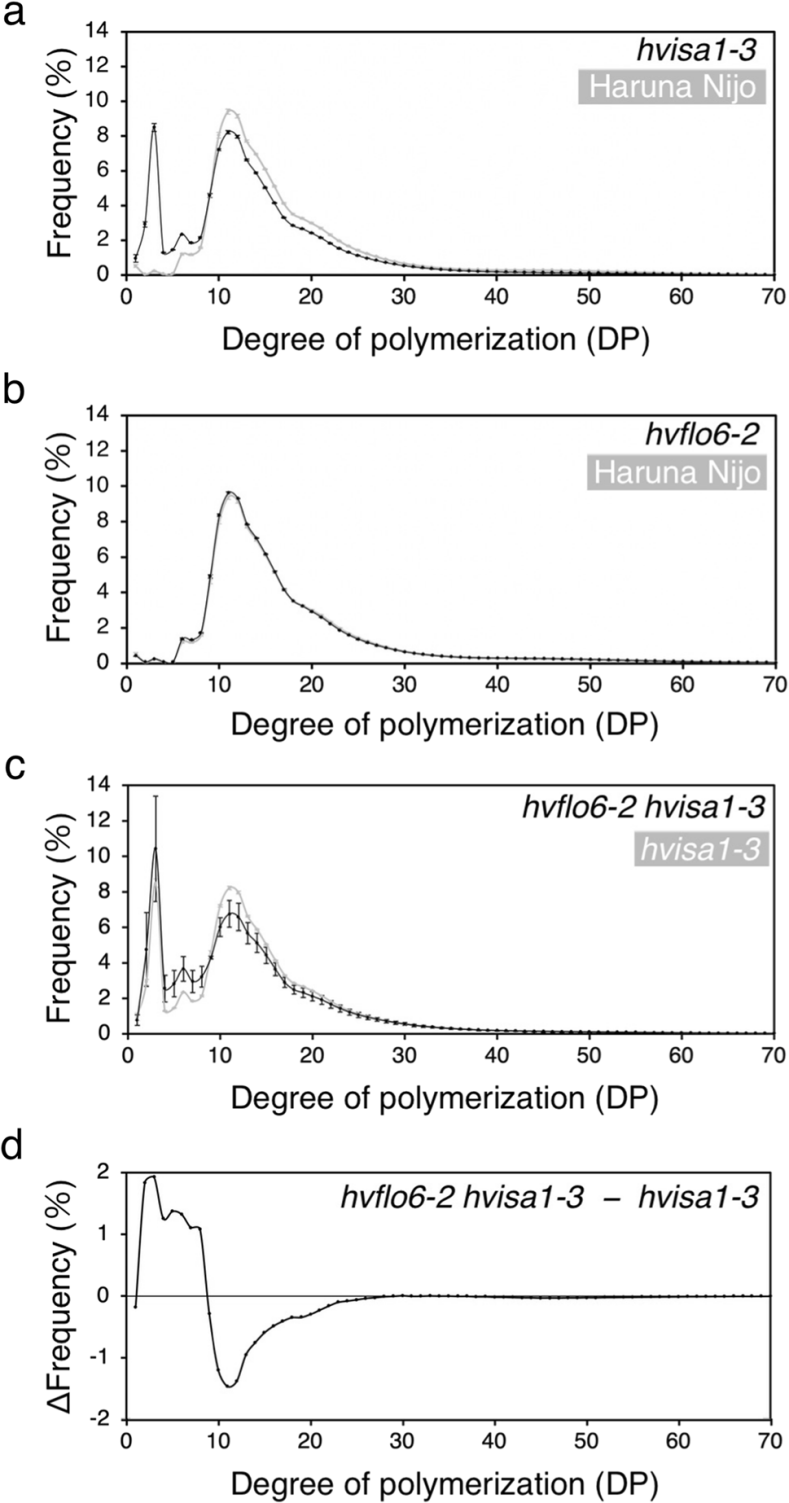
Glucose chain-length distribution of α-glucans in mutant grains. **a** *hvisa1-3* and Haruna Nijo are indicated by black and grey lines, respectively. **b** *hvflo6-2* and Haruna Nijo are indicated by black and grey lines, respectively. **c** *hvflo6-2 hvisa1-3* and *hvisa1-3* are indicated by black and grey lines, respectively. Data are given as means ± SD. All data were obtained from at least three independent grains. **d** Difference plot corresponding to the chain-length distribution profile presented in (c). The value for each chain length from the *hvisa1-3* was subtracted from that of *hvflo6-2 hvisa1-3*

In various *isa1* mutants, the distribution of chain lengths of total α-glucans is altered compared to parental lines, with more short glucose chains and fewer long glucose chains (Zeeman et al. 1998; Kubo et al. 1999; Dinges et al. 2001; Burton et al. 2002). These alterations are mainly due to an increase in highly branched phytoglycogen (Fujita et al. 2003). We speculated that chain-length distributions of total α-glucans might also be affected in *hvflo6-2 hvisa1-3* (Fig. 6)*.* In *hvisa1-3* endosperm, shorter glucose chains (degree of polymerization [DP] < 10) were more abundant and longer glucose chains (DP > 10) were less abundant than in Haruna Nijo (Fig. 6a). A similar pattern was observed in the *hvisa1-5* mutant compared to the parental line, Morex (Supplementary Fig. 12a). In *hvflo6-2,* the chain-length distribution was similar to that of Haruna Nijo and the *hvflo6-1* mutant (Fig. 6b, Supplementary Fig. 12b). Thus, the *hvflo6* mutations had no major effect on α-glucan structure. The shift from longer to shorter chain lengths seen in *hvisa1-3* was even more pronounced in *hvflo6-2 hvisa1-3* (Fig. 6c, d). This result is consistent with the increased amount of phytoglycogen in *hvflo6-2 hvisa1-3.*

### Sugar levels in hvflo6-2 hvisa1-3 grain

**Fig. 7:**
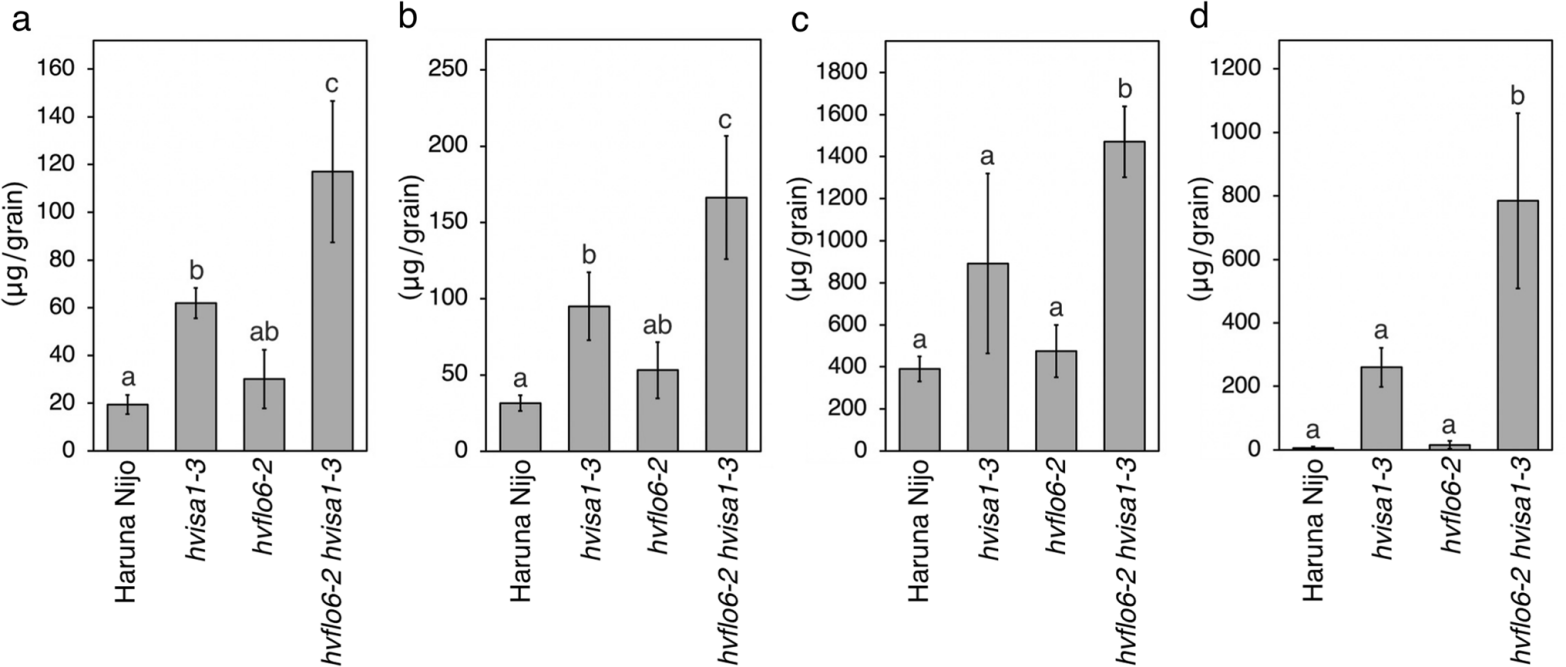
Elevated sugar accumulation in *hvflo6-2 hvisa1-3*. Sugars in mature grains of the wild type and the single and double mutants were quantified using GC-MS (*n* = 4). **a** Glucose. **b** Fructose. **c** Sucrose. **d** Maltose. Data are given as means ± SD. Statistical comparisons were performed using Tukey’s HSD. The same letters above the bars represent statistically indistinguishable groups, and different letters represent statistically different groups (*p* < 0.05)

Starch accumulation in *hvflo6-2 hvisa1-3* mature grain was approximately 21% that of Haruna Nijo (Fig. 2h). Starch accumulation often negatively correlates with sugar accumulation (Shannon et al. 1996; Schreier et al. 2021). Higher sugar accumulation is a characteristic trait of some *isa1* mutants (Gonzales et al. 1976; Pan and Nelson 1984; James et al. 1995). We quantified glucose, fructose, sucrose, and maltose in mature grains of mutants (Fig. 7). In *hvisa1-3*, glucose and fructose accumulated to higher levels than in Haruna Nijo and *hvflo6-2* (Fig. 7a, b)*.* Sucrose and maltose also had greater accumulation in *hvisa1-3* than in Haruna Nijo, although the increase was not statistically significant (Fig. 7c, d). There were no statistically significant differences between *hvflo6-2* and Haruna Nijo in the accumulation of any of the four sugars. All four sugars accumulated to higher levels in *hvflo6-2 hvisa1-3* than in *hvisa1-3.*

ADP-glucose is the substrate of SS and the glucosyl donor for starch biosynthesis. ADP-glucose content is known to increase in low-starch mutants in several plant species. (Seung et al. 2016; Crofts et al. 2022a; Watson-Lazowski et al. 2022). So, we quantified the ADP-glucose in the low-starch *hvflo6-2 hvisa1-3* mutant. ADP-glucose significantly increased in the developing endosperm of *hvflo6-2 hvisa1-3* compared to Haruna Nijo (Supplementary Fig. 13).

### Starch biosynthetic enzyme activities in mutants

**Fig. 8:**
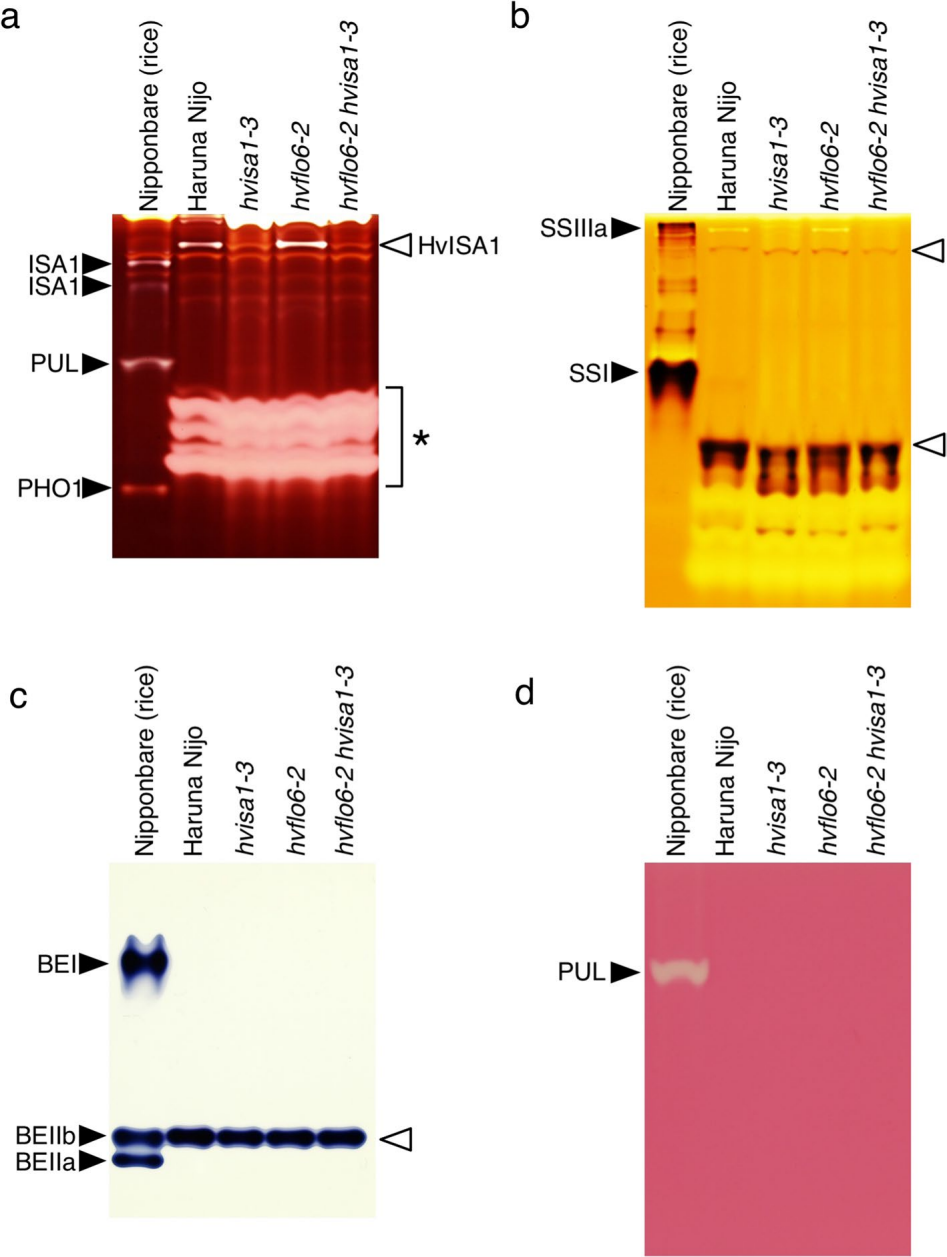
Native-PAGE activity staining of starch biosynthetic enzymes in mutants. **a** Native-PAGE debranching enzyme (DBE) activity staining. Rice ISA1, PUL, PHO1 activity bands are indicated by black arrowheads. HvISA1 activity bands are indicated by a white arrowhead. Unknown glycosyl hydrolase bands are indicated with an asterisk. **b** Native-PAGE starch synthase (SS) activity staining. Rice SSIIIa and SSI activity bands are indicated by black arrowheads. SS activity bands derived from barley are indicated by white arrowheads. **c** Native-PAGE branching enzyme (BE) activity staining. Rice BEI, BEIIa and BEIIb activity bands are indicated by black arrowheads. BE activity bands derived from barley are indicated by a white arrowhead. **d** Native-PAGE pullulanase (PUL) activity staining. Rice PUL activity band is indicated by a black arrowhead. PUL activity bands were not detected from developing barley grains. Activity bands of rice were assigned according to Fujita et al. (2009) and Crofts et al. (2022b)

To investigate starch-related enzymatical activities in the mutants, we performed native-PAGE activity staining of SS, BE, DBE, and PUL using developing grains. PUL is another type of DBE than ISA1. PUL hydrolyzes α-1,6 linkages of amylopectin and pullulan, unlike ISA1 hydrolyzes amylopectin and glycogen but not pullulan (Nakamura 1996). We used proteins from developing rice grains as a positive control, and activity bands from rice were assigned according to the previous reports (Fujita et al. 2009; Crofts et al. 2022b). The DBE activity staining detected ISA1, PUL, and plastidial phosphorylase (PHO1) bands from rice grains (Fig. 8a). Barley HvISA1 was detected in Haruna Nijo and *hvflo6-2* but not in *hvisa1-3* and *hvflo6-2 hvisa1-3* (Fig. 8a). This result is consistent with the accumulation level of HvISA1 proteins (Supplementary Fig. 4). SS activity was detected as SSI and SSIIIa bands in rice grains (Fig. 8b). In barley, two bands were detected in Haruna Nijo and mutants, however there was no significant difference in the band intensities among them. The upper band with slightly faster mobility than rice SSIIIa might be HvSSIIIa, while the lower band might be HvSSI showing the major SS activity in barley. BE activity was detected as three bands of BEI, BEIIa, and BEIIa in rice. In contrast, a single band was detected in barley (Fig. 8c). This should be the overlapped bands of HvBEIIa and HvBEIIb according to the previous activity staining study (Regina et al. 2010). The band with BE activity showed almost the same intensity among Haruna Nijo and mutants. PUL activity band was detected as a single band of PUL in rice but not from barley (Fig. 8d).

## Discussion

### Isolation of allelic mutants of HvISA1 and HvFLO6 genes

In this study, we isolated allelic mutants of the *HvISA1* and *HvFLO6* from barley (Fig. 1, Supplementary Fig. 1). The *hvisa1-3* and *hvflo6-2* single mutants are both in the Haruna Nijo genomic background, allowing us to study their genetic interaction without the complications of different genetic backgrounds. The *hvisa1-3* mutant had a premature stop codon. The predicted truncated HvISA1 protein (489 aa) lacks one of the catalytic triad residues, several substrate binding residues, and the dimerization domain (Sim et al. 2014). HvISA1 protein did not accumulate in *hvisa1-3* (Fig. 1k, Supplementary Fig. 4). The *hvflo6-2* mutant also had a premature stop codon. The predicted truncated protein (216 aa) lacks the coiled-coil region and the crucial CBM48 domain. These results suggest that *hvisa1-3* and *hvflo6-2* are null mutations.

The *hvisa1-3* and *hvflo6-2* single mutants showed increased numbers of compound SGs in the endosperm (Fig. 1f, h) and *hvflo6-2* also showed increased numbers of compound SGs in pollen grains; this phenotype was more pronounced in *hvflo6-2 hvisa1-3* double mutants (Fig. 4). This means that HvISA1 and HvFLO6 are involved in starch biosynthesis in endosperm and in pollen grains. Seed setting on panicles of *hvflo6*-2 and *hvflo6-2 hvisa1-3* was not affected (Fig. 2a, e). Therefore, the increase in compound SGs in pollen may not be associated with pollen viability.

We lack a molecular explanation for the increased compound SGs in *hvisa1* and *hvflo6* mutants. It is hypothesized that the soluble phytoglycogen molecules or their degradation products, such as maltooligosaccharides, may serve as suitable substrates for granule initiation in *isa1* mutants (Seung and Smith 2019). Phytoglycogen levels were increased significantly in *hvisa1-3* but not in *hvflo6-2* (Fig. 5A). HvFLO6 is suggested to restrict the initiation of B-type granules in plastids during the early developmental stage, and loss of HvFLO6 can increase the B-type granule initiations in plastids leading to the compound SG formation (Chia et al. 2020). In pollen, the increased compound SGs was only observed in *hvflo6-2* but not in *hvisa1-3* (Fig. 4). Therefore, it is likely that the function of HvFLO6 do not overlap with that of HvISA in pollen.

SGs in *hvisa1-3* pollen were indistinguishable from those in wild-type pollen (Fig. 4). So, HvISA1 may not be necessary for starch biosynthesis in pollen. The biochemical function of ISA1 is to remove glucose chains created at inappropriate positions of amylopectin. On the other hand, BE has the activity to create branches in amylopectin. Therefore, the necessity for ISA1 should be lower in the tissue with low BE activity. In fact, the rice *isa1* mutant phenotype is alleviated in both *beIIa* and *beIIb* mutant backgrounds (Lee et al. 2017; Nagamatsu et al. 2022). Rice *BEIIa* and *BEIIb* genes are expressed in pollen, but the expression levels are considerably lower than the *BEIIb* major expression in the endosperm (https://ricexpro.dna.affrc.go.jp/index.html, Sato et al. 2013). The lower level of BE expression in pollen compared to the endosperm may reduce the requirement for ISA1 in pollen.

### The hvisa1 and hvflo6 mutations mutually enhanced each other’s phenotype

The *hvflo6-2 hvisa1-3* double mutant grains accumulated higher levels of phytoglycogen and sugars than did *hvisa1-3* grains (Figs. 5a, 7). Furthermore, the chain-length distribution of total α-glucans was more severely affected in *hvflo6-2 hvisa1-3* than in *hvisa1-3* (Fig. 6). The *hvflo6-2* single mutant did not show significant accumulations of phytoglycogen and sugars compared to the wild type (Figs. 5a, 7). Therefore, *hvflo6-2* enhances the *hvisa1-3* mutation in barley; this effect on *hvisa1-3* was also observed with another allele, *hvflo6-1* (Supplementary Fig. 11)*.* In contrast, increased compound SG in pollen was observed in *hvflo6-2* but not in *hvisa1-3*, and the pollen phenotype was enhanced in *hvflo6-2 hvisa1-3* double mutant (Fig. 4). This suggests both mutations mutually enhance each other’s phenotypes. ISA1 and FLO6 are shown to interact physically in rice (Peng et al. 2014), and it will be necessary to determine how the interaction is related to the enhancing phenotypes in future studies.

Other mutations that affect *isa1* phenotypes include defects in starch biosynthesis genes, such as *SSIIa*, *PUL*, *BEIIa* and *BEIIb*. Rice *isa1* mutant from japonica variety with less SSIIa activity showed more severe *isa1* phenotype than indica *isa1* mutant with active SSIIa (Crofts et al. 2022b). *pul* mutation in *isa1* background showed elevated level of phytoglycogen and reduced starch accumulation in rice, maize and Arabidopsis (Dinges et al. 2003; Wattebled et al. 2005; Fujita et al. 2009). In contrast, *beIIa* and *beIIb* mutations mitigate the *isa1* phenotype in rice (Lee et al. 2017; Nagamatsu et al. 2022). We could not detect significant difference in enzymatic activities of SS, BE among mutants in this study (Fig. 8b, c). PUL activity band was not detected in barley under the condition where rice PUL activity band was detected (Fig. 8d). This suggests that barley’s PUL activity during grain development is not as high as in rice. We cannot exclude the possibility that undetectable isozymes in our native-PAGE activity staining are involved in the *hvisa1* phenotypic enhancement. Some barley enzymes showed different band mobility from rice orthologs, which made it difficult to identify the isozymes for each activity band in barley. Different charge status or protein complexes may be attributed to the mobility difference. It will be necessary to see which bands are derived from which isozymes in barley using each isozyme mutant.

The loss of ISA1 by maize *sugary1* mutation causes the sweet kernel phenotype because of its high sugar accumulation (Gonzales et al. 1976; Pan and Nelson 1984; James et al. 1995). In maize, an enhancer mutant for the sugary phenotype was isolated as *sugary enhancer 1* (*se1*) (Ferguson et al. 1979). *SE1* encodes a 173-aa protein containing a FANTASTIC FOUR domain (Zhang et al. 2019). Phylogenetic analysis revealed that *SE1* orthologs are present in monocots but not dicots. Mature kernels of the *sugary1 se1* double mutant accumulate higher levels of sugars and phytoglycogen compared to the *sugary1* single mutant. The mechanism by which SE1 interacts with starch metabolism and ISA1 function is unknown.

Recently, a maize ortholog of *HvFLO6*, *ZmCBM48-1*, was identified (Peng et al. 2022). Overexpression of *ZmCBM48-1* in rice increased the starch and reduced soluble sugar content. ZmCBM48-1 likely plays a positive regulatory role in the starch biosynthesis pathway by up-regulating several starch biosynthesis genes. Maize mutants defective in *ZmCBM48-1* have not been isolated. Because the sugary enhancer phenotype is useful in the sweet corn market, it would be worthwhile to isolate a *zmcbm48-1* mutant and determine whether it acts as a sugary enhancer in maize.

### HvFLO6 function in starch biosynthesis

*FLO6* is a rice ortholog of *HvFLO6* (Peng et al. 2014). The amino acid sequences of HvFLO6 and FLO6 are more than 60% identical and *FLO6* is the most closely related gene to *HvFLO6* in the rice genome. FLO6 interacts with ISA1 in rice and is suggested to act as a scaffold mediating the binding of ISA1 to starch. However, *isa1* has a more severe phenotype than *flo6* in rice and barley, suggesting that FLO6 is not always required for ISA1 function. Consistent with this notion, the HvISA1 band of *hvflo6-2* in the native-PAGE activity staining was detected as wild type (Fig. 8a). So far, no double mutants of *flo6* and *isa1* mutants have been reported in rice. Therefore, *hvflo6-2 hvisa1-3* double mutant is an ideal material to study the genetic interaction between these mutations. We showed that the *hvflo6-2 hvisa1-3* double mutant had more severe phenotypes than *hvisa1-3*, suggesting that in barley, HvFLO6 has additional functions beyond supporting HvISA1.

Starch biosynthetic enzymes are reported to form protein complexes to coordinate the reactions of starch biosynthesis in rice, wheat, barley, and maize (Tetlow et al. 2004; Hennen-Bierwagen et al. 2008; Ahmed et al. 2015; Crofts et al. 2018; Ida et al. 2022). The active enzyme complex formation may be mediated by PTST/ FLO6 proteins. Arabidopsis PTST2 is shown to interact with SS4, and rice FLO6 binds to ISA1, SSIVb and granule-bound starch synthases (GBSSI and GBSSII) (Seung et al. 2017; Zhang et al. 2022). HvFLO6 may also mediate active protein complex formation by interacting with starch biosynthetic enzymes. *B-GRANULE CONTENT1* (*BGC1*), wheat homolog of *HvFLO6,* is shown to promote the initiation of B-type granules in plastids in the later grain development (Chia et al. 2020). While, in the early grain development, *BGC1* restricts the initiation of B-type SGs so that A-type SGs form in the main body of the plastid. The stage-dependent opposite function of BGC1 may attribute to the stage-dependent different binding partners. SSs, BEs, and DBEs have overlapping expression timing but peak at different developmental stages in rice and barley (Ohdan et al. 2005; Radchuk et al. 2009). It is possible that FLO6 orthologs bind to different starch biosynthetic enzymes in developmental stage specific manner.

### Which materials accumulate in hvflo6 hvisa1 grain?

As shown in Fig. 2, the double mutant had less starch in the developing and mature grains. In this study, we quantified the amounts of starch, phytoglycogen, β-glucan, and sugars in mature grains of barley mutants. In Haruna Nijo, *hvisa1-3*, and *hvflo6-2*, starch accounted for 67, 53, and 59%, respectively, of the single grain weight. In *hvflo6-2 hvisa1-3*, starch accounted for only 19% of the grain weight (Supplementary Fig. 14). The remainder of the grain, which includes the embryo, seed coat, and other materials, accounted for 23% (about 12 mg), 32% (about 15 mg), 30% (about 14 mg), and 56% (about 23 mg) of the grain weight in Haruna Nijo, *hvisa1-3*, *hvflo6-2*, and *hvflo6-2 hvisa1-3*, respectively. The weights of these remaining parts of the grain were similar in Haruna Nijo, *hvisa1-3*, and *hvflo6-2*, ranging from 12 to 15 mg. Assuming that this value is common to all barley grains and thus can be applied to *hvflo6-2 hvisa1-3*, we subtracted this value from the amount of remaining material in the double mutant grains. This calculation showed that the double mutant grains had 8–11 mg/grain of undetermined materials that were not present in Haruna Nijo or the single mutants.

In Chlamydomonas, *isa1* mutants have reduced starch and increased phytoglycogen contents, resulting in altered diurnal carbohydrate metabolism (Kato et al. 2021). This disruption can lead to high accumulations of lipids and carotenoids inside the cells. Defects in starch biosynthesis also enhance lipid biosynthesis in Arabidopsis leaves (Yu et al. 2018). Therefore, the undetermined materials in *hvflo6-2 hvisa1-3* grains may be lipid-related. Identification of the substances that accumulate in *hvflo6-2 hvisa1-3* grains instead of starch will require further analysis.

Starch amounts have been reported to negatively correlate with β-glucan amounts in barley grains (Islamovic et al. 2013; Shu and Rasmussen 2014). However, β-glucan was not increased in *hvflo6-2 hvisa1-3* (Fig. 5b), perhaps because the parental line, Haruna Nijo, is an elite malting variety selected for reduced β-glucan levels during breeding. *hvflo6-2 hvisa1-3* might display an increased β-glucan phenotype in the genetic background of a non-malting variety.

The mutants isolated in this study are allelic variations of existing mutants, but we expanded the phenotype by creating the double mutant. Expanding starch-related phenotypes by multiplexing existing mutants will be important in breeding plant varieties with desirable starch properties. Barley is a versatile crop used for malting, food, and feed, and grain starch properties are an important determinant of barley’s suitability for these different applications. Continued screening based on SG morphology will allow the isolation of novel mutants that might advance barley breeding.

## Supplementary Information

Below is the link to the electronic supplementary material.

Supplementary file1 (PDF 4080 KB)

## Data Availability

The sequences of *HvISA1* and *HvFLO6* are available in the GrainGenes database (https://wheat.pw.usda.gov) as HORVU.MOREX.r2.7HG0565540.1 and HORVU.MOREX.r2.4HG0279220.1, respectively.

## Abbreviations

DAA: Days after awn emergence
*HvISA1*: *Hordeum vulgare ISOAMYLASE1*
*HvFLO6*: *Hordeum vulgare FLOURY ENDOSPERM 6*
SG: Starch granule
