# Supplementary material for: FLOURY ENDOSPERM 6 mutations enhance the sugary phenotype caused by the loss of ISOAMYLASE1 in barley
Source: Theor Appl Genet. 2023 Apr 3;136(4):94. doi: 10.1007/s00122-023-04339-5 (PMC10070237; doi:10.1007/s00122-023-04339-5)
Supplement: Supplementary file 1 — Supplementary file1 (PDF 4080 KB) [file 122_2023_4339_MOESM1_ESM.pdf]

Supplementary Fig. 1

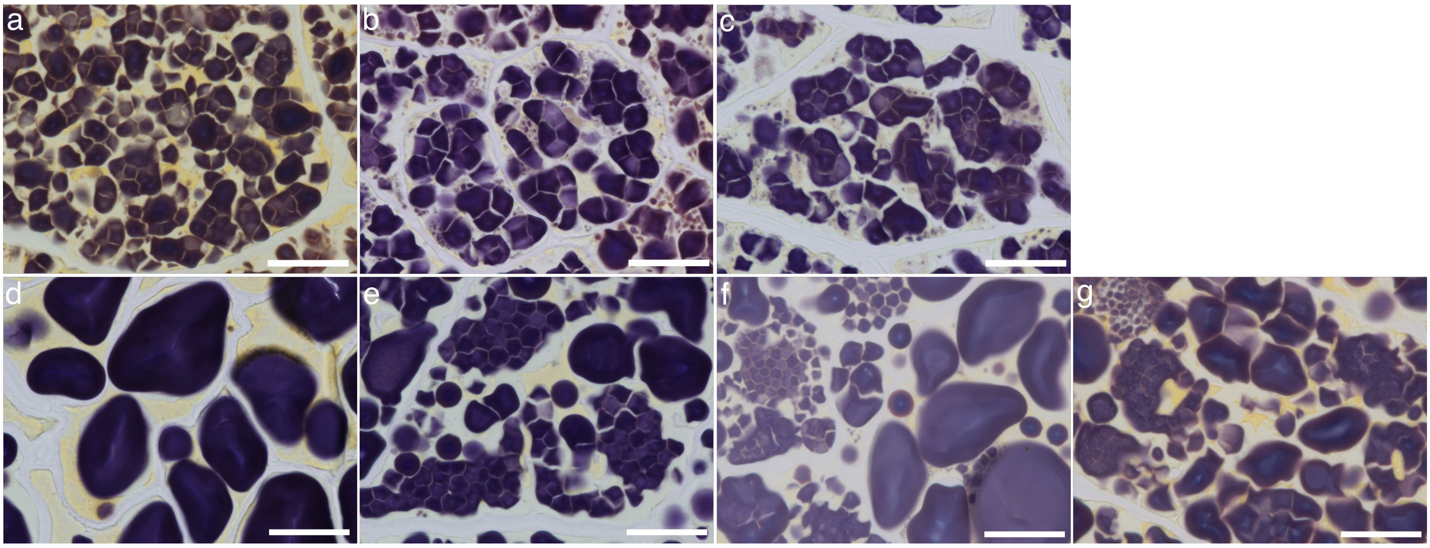

Supplementary Fig. 1 SGs in different mutants of *hvisa1* and *hvflo6*.
a–c Iodine-stained thin sections of endosperm cells of *hvisa1-4* (a), *hvisa1-5* (b), and *Risø17* (*hvisa1-1*) (c). **d, e** Iodine-stained thin sections of endosperm cells of *Franubet* (*hvflo6-1*). **f, g** Iodine-stained thin sections of endosperm cells of F1 grains from a cross between *hvflo6-2* and *Franubet* (*hvflo6-1*). Bars = 20 µm.

Supplementary Fig. 2

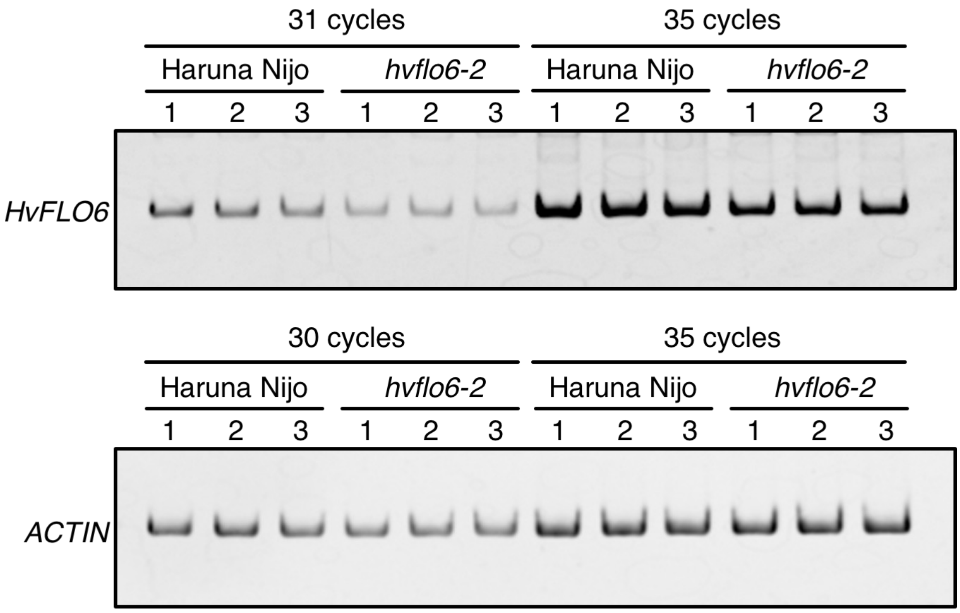

Supplementary Fig. 2 Expression of *HvFLO6*.
Reverse transcription (RT)-PCR to check the expression of *HvFLO6* in individual 15 DAA grains of Haruna Nijo and *hvflo6-2*. Total RNA was isolated from three independent grains, and cDNA was amplified with two different PCR cycles. *ACTIN* was used as the internal control.

Supplementary Fig. 3

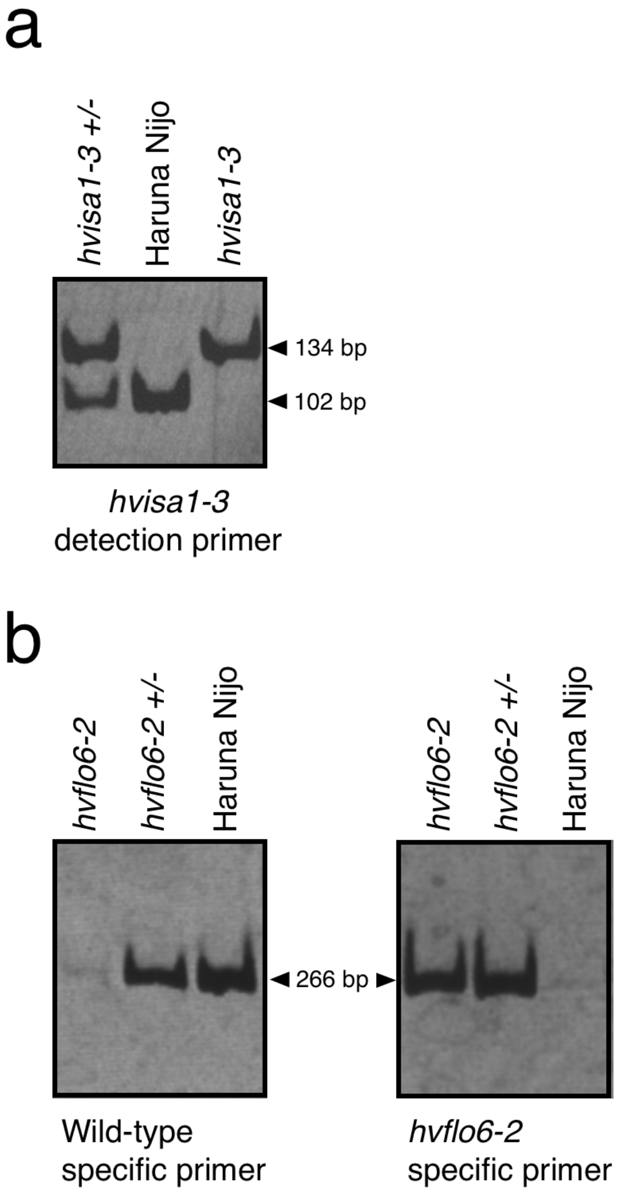

Supplementary Fig. 3 PCR detection of base changes in the *hvisa1-3* and *hvflo6-2* mutants. **a** To detect the *hvisa1-3* mutation, PCR products were amplified using derived cleaved-amplified polymorphic sequence primers and digested with *Hinf*I. In the case of the wild-type allele (Haruna Nijo), the PCR product (134 bp) was digested into fragments of 102 bp and 32 bp (the smaller band is not visible). In the case of the *hvisa1-3* allele, the PCR product was not digested. Heterozygous plants (*hvisa1-3* $^{+/-}$) yielded both the full-length band and the digestion products. **b** To detect the *hvflo6-2* mutation, PCR products were amplified with two different primer sets, one specific for the wild-type allele and the other specific for the *hvflo6-2* allele. The wild-type-specific primer set amplified a 266-bp PCR product from the wild-type allele (Haruna Nijo) but not from the *hvflo6-2* allele. In contrast, the *hvflo6-2*-specific primer pairs amplified a 266-bp PCR product from the *hvflo6-2* allele but not the wild-type allele. Both primer pairs yielded bands from heterozygous samples.

Supplementary Fig. 4

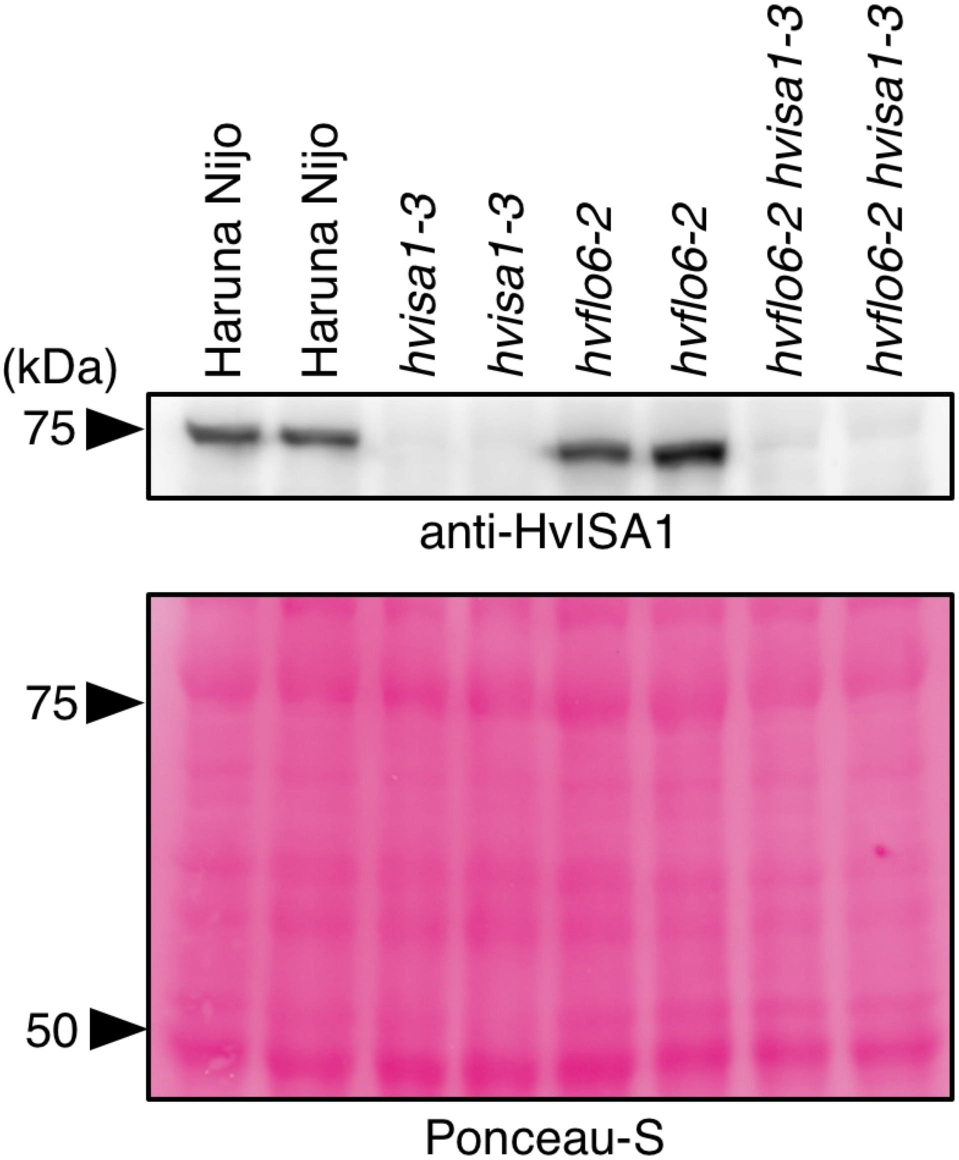

Supplementary Fig. 4 No accumulation of HvISA1 in *hvflo6-2 hvisa1-3*.
Immunoblot analysis using anti-HvISA1 antisera (top). The Ponceau S-stained membrane is also shown (bottom). HvISA1 accumulated in Haruna Nijo and *hvflo6-2* but not in *hvisa1-3* or *hvflo6-2 hvisa1-3*.

Supplementary Fig. 5

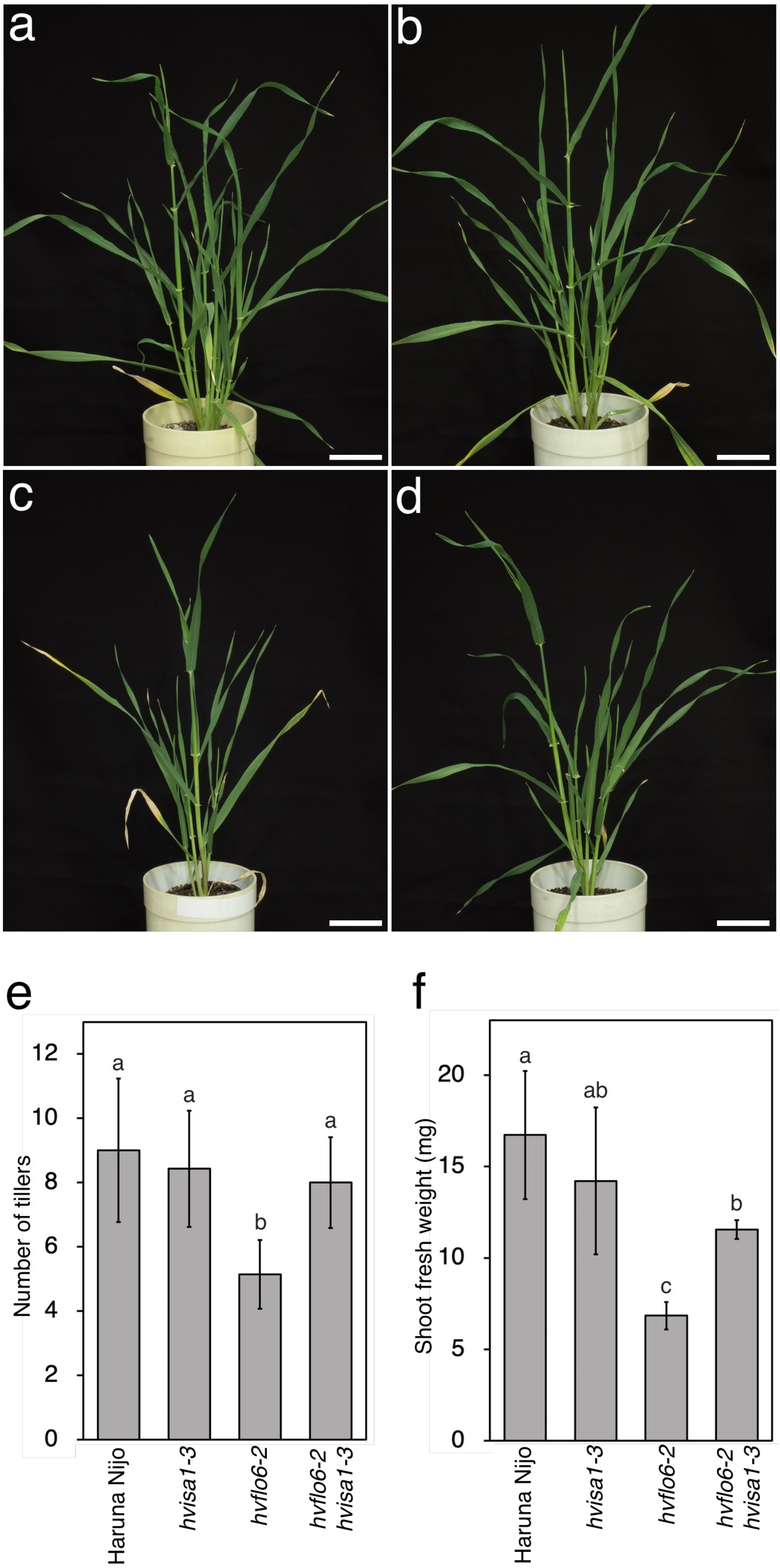

Supplementary Fig. 5 Growth phenotype of the mutants.
a–d Photographs of 25-day-old plants of Haruna Nijo (a), *hvisa1-3* (b), *hvflo6-2* (c), and *hvflo6-2 hvisa1-3* (d). Bars = 5 cm. **e** The number of tillers of 25-day-old plants (n = 7). **f** Shoot fresh weight of 25-day-old plants (n = 5–6). Data are given as means ± SD. Statistical comparisons were performed using Tukey's HSD. The same letters above the bars represent statistically indistinguishable groups, and different letters represent statistically different groups ($p < 0.05$).

Supplementary Fig. 6

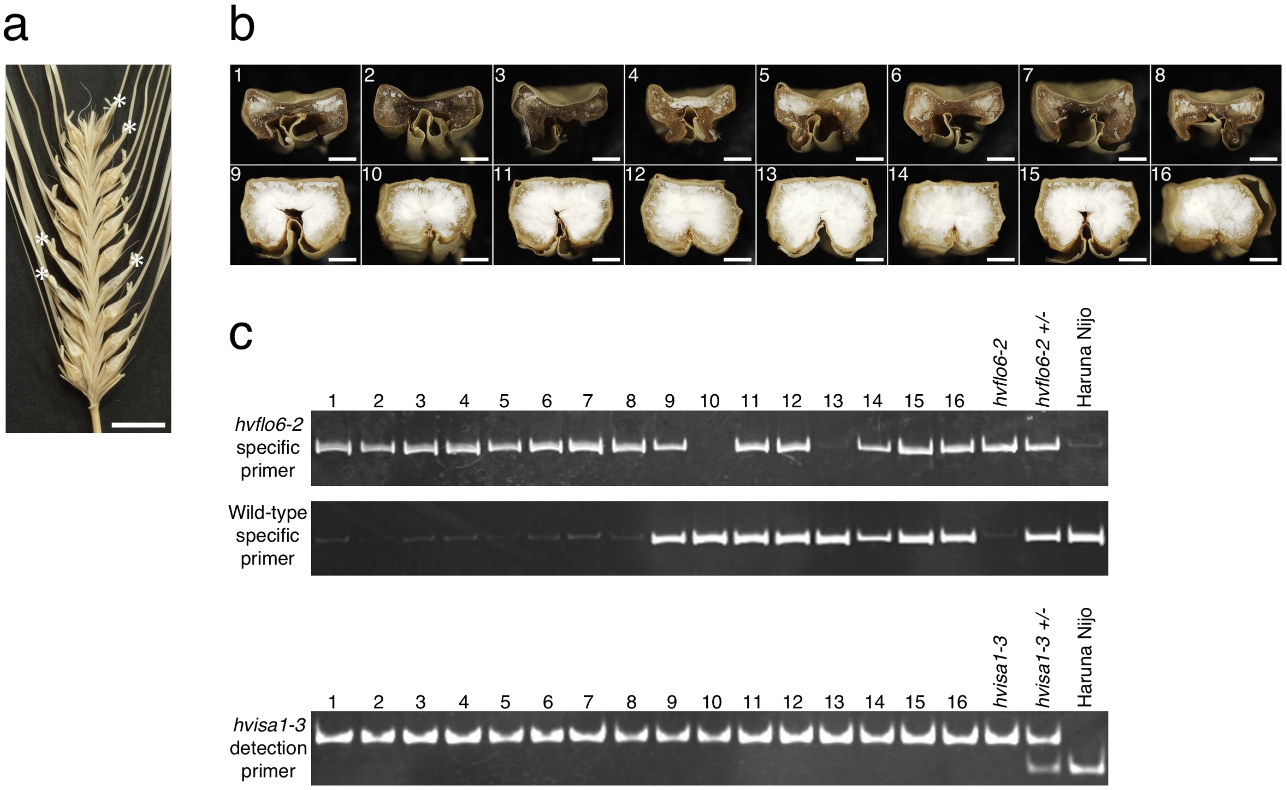

Supplementary Fig. 6 Co-segregation of the *hvflo6-2 hvisa1-3* genotype with the shrunken grain phenotype.

a A mature panicle of a *hvflo6-2*$^{+/-}$ *hvisa1-3* plant. Asterisks indicate shrunken grains. Bar = 1 cm. **b** Cross-sections of shrunken grains (1–8) and normal grains (9–16) obtained from a self-fertilized *hvflo6-2*$^{+/-}$ *hvisa1-3* plant. Bars = 1 mm. **c** Genotyping of the grains in (b) using primers designed to detect the *hvflo6-2* and *hvisa1-3* alleles. Lane numbers match the grain numbers. All eight shrunken grains were homozygous mutants at both loci.

Supplementary Fig. 7

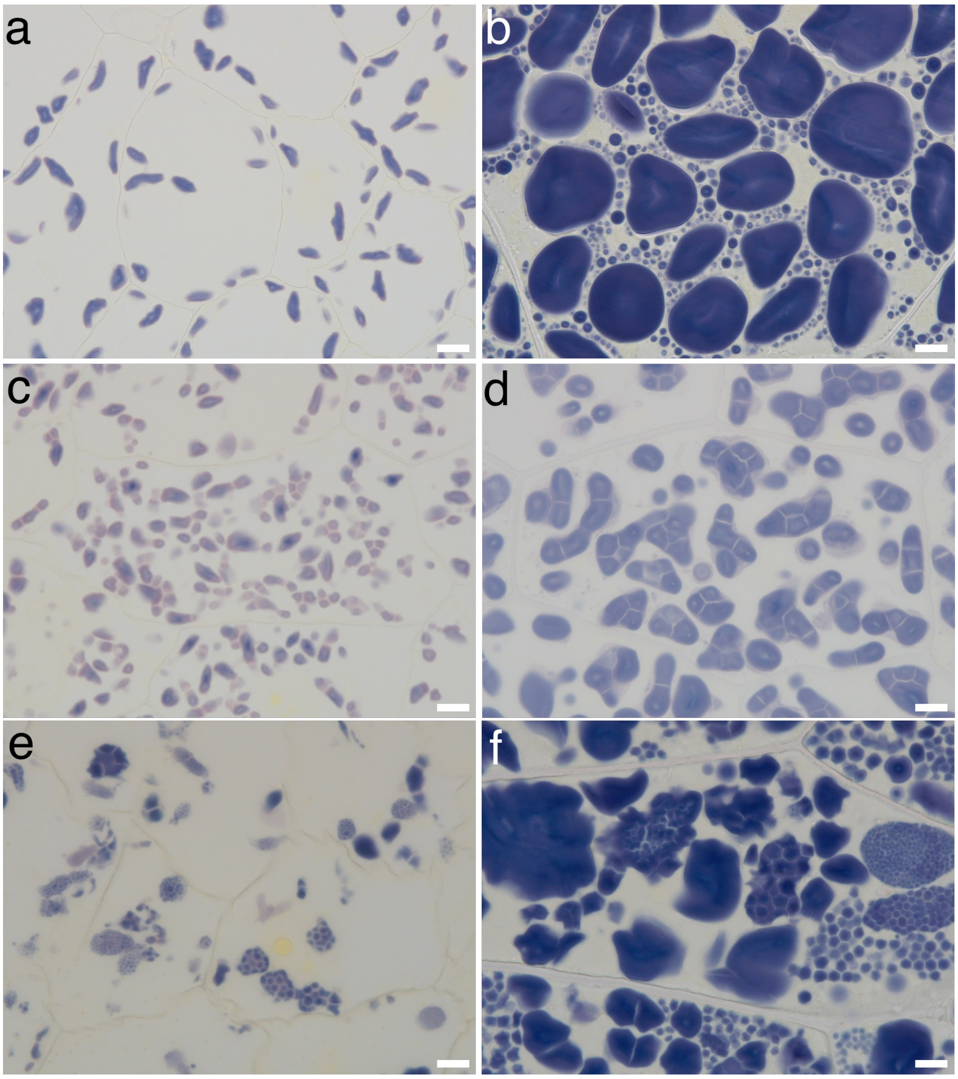

Supplementary Fig. 7 SGs in developing grains at 10 DAA and 20 DAA.
a, **b** Iodine-stained thin sections of endosperm cells of Haruna Nijo at 10 DAA (a) and 20 DAA (b). **c**, **d** Iodine-stained thin section of endosperm cells of *hvisa1-3* at 10 DAA (c) and 20 DAA (d). **e**, **f** Iodine-stained thin section of endosperm cells of *hvflo6-2* at 10 DAA (e) and 20 DAA (f). Bars = 10 μm.

Supplementary Fig. 8

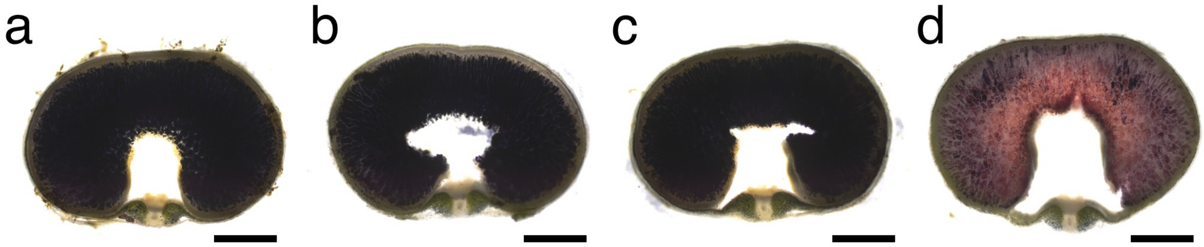

Supplementary Fig. 8 Grain cross-sections stained with iodine without washing with water. Cross-sections of grains at 20 DAA were stained in iodine solution on a glass slide and observed immediately. **a** Haruna Nijo. **b** *hvisa1-3*. **c** *hvflo6-2*. **d** *hvflo6-2 hvisa1-3*. Bars = 1 mm.

Supplementary Fig. 9

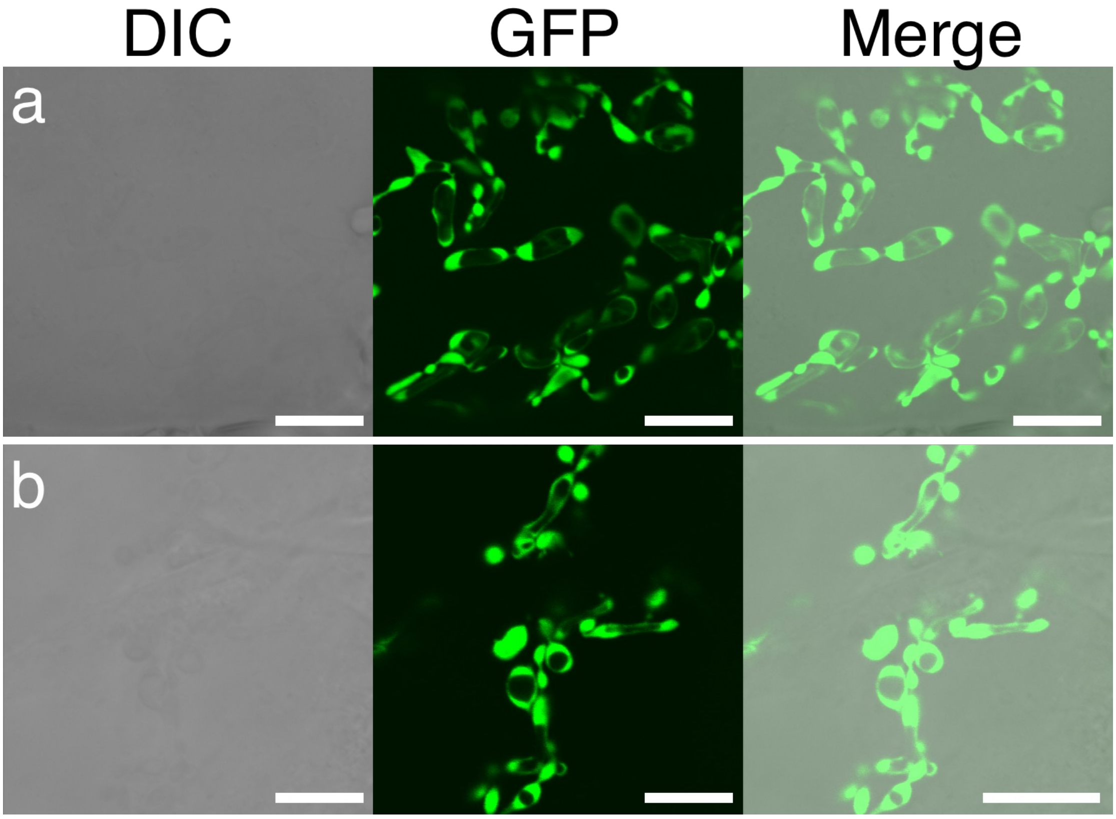

Supplementary Fig. 9 Fluorescence images of GFP-labeled amorphous amyloplasts.
a Differential interference contrast (DIC), GFP, and merged images of amorphous amyloplasts in *hvflo6-2* endosperm at 15 DAA. **b** Amorphous amyloplasts in *hvflo6-2 hvisa1-3* endosperm at 15 DAA. Bars = 10 μm.

Supplementary Fig. 10

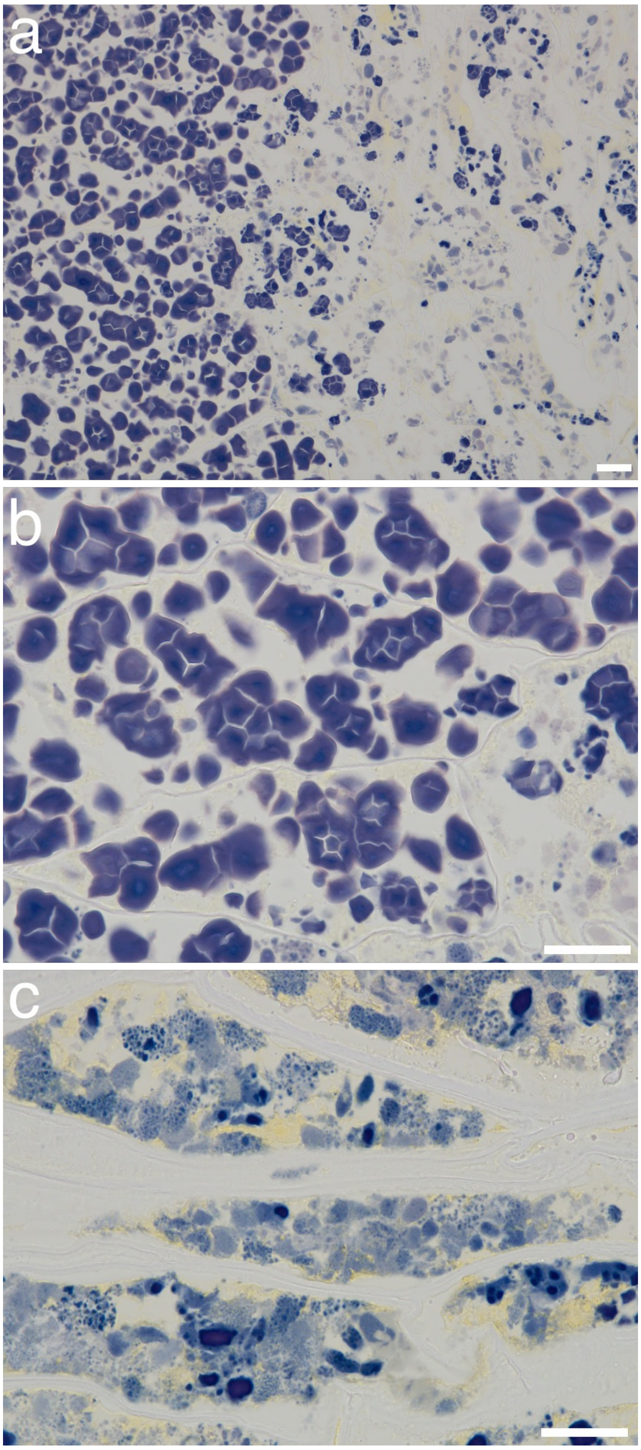

Supplementary Fig. 10 SGs in mature grains of *hvflo6-2 hvisa1-3*.
a Iodine-stained thin section of *hvflo6-2 hvisa1-3* endosperm at mature stage. **b** Enlarged view of compound SGs in the starch-rich region. **c** Enlarged view of defective SGs in the starch-less region. Bars = 20 μm.

Supplementary Fig. 11

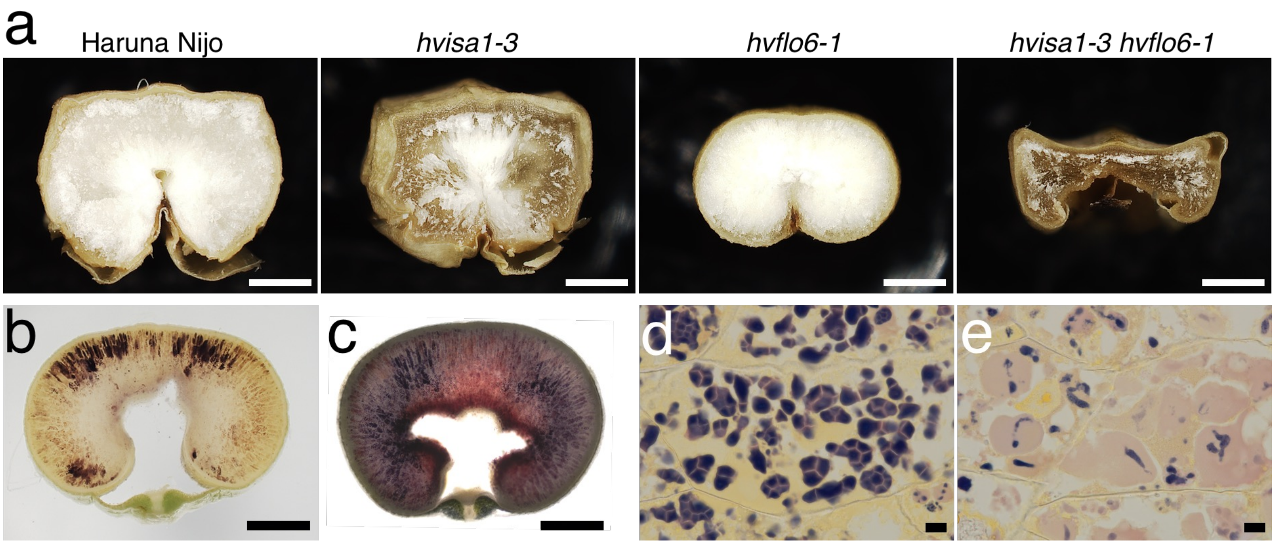

Supplementary Fig. 11 Phenotypes of *hvisa1-3 hvflo6-1*.
a Unstained cross-sections of mature grains of Haruna Nijo, *hvisa1-3*, *hvflo6-1*, and *hvisa1-3 hvflo6-1*. **b** Iodine-stained cross-sections of *hvisa1-3 hvflo6-1* grains at 20 DAA with washing. Bar = 1 mm. **c** Iodine-stained cross-sections of *hvisa1-3 hvflo6-1* grains at 20 DAA without washing. Bar = 1 mm. **d** Compound SGs in a starch-rich region of *hvisa1-3 hvflo6-1* endosperm at 15 DAA. Bar = 10 µm. **e** Pinkish balloon-like structures in a starch-less region of *hvisa1-3 hvflo6-1* endosperm at 15 DAA. Bar = 10 µm.

Supplementary Fig. 12

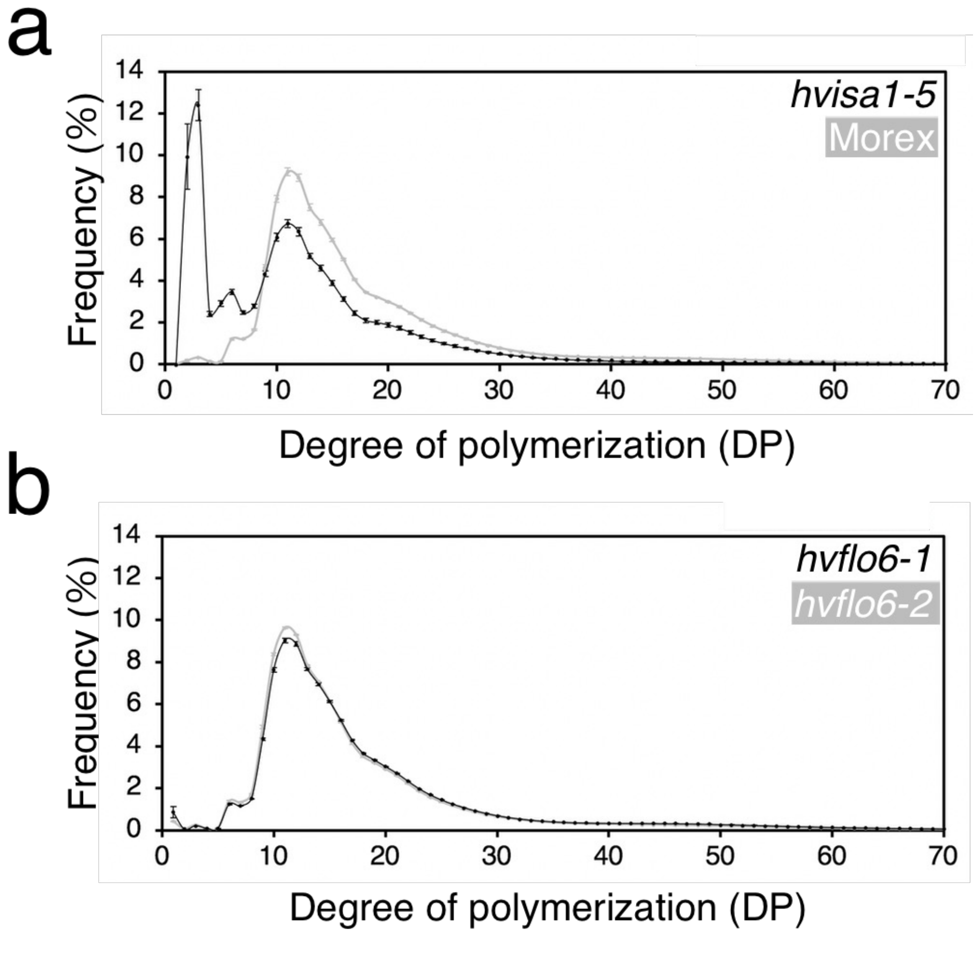

Supplementary Fig. 12 Glucose chain-length distribution analysis of α-glucans of *hvisa1-5* and *hvflo6-1*.

a *hvisa1-5* and Morex are indicated by black and grey lines, respectively. **b** *hvflo6-1* and *hvflo6-2* are indicated by black and grey lines, respectively. Data are given as means ± SD. All data were obtained from at least three independent grains.

Supplementary Fig. 13

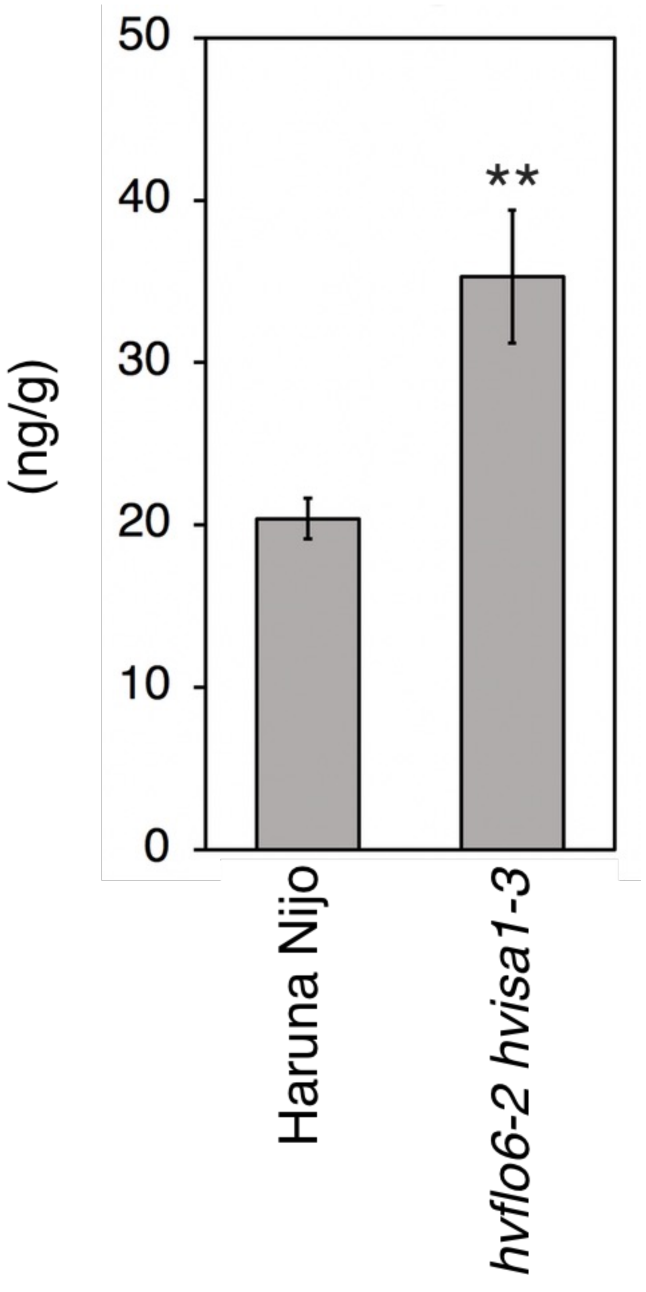

Supplementary Fig. 13 Elevated accumulation of ADP-glucose in *hvflo6-2 hvisa1-3*. Data are given as means ± SD (n = 3). Statistical comparison was performed using Student's *t*-test (**$p <$ 0.01).

Supplementary Fig. 14

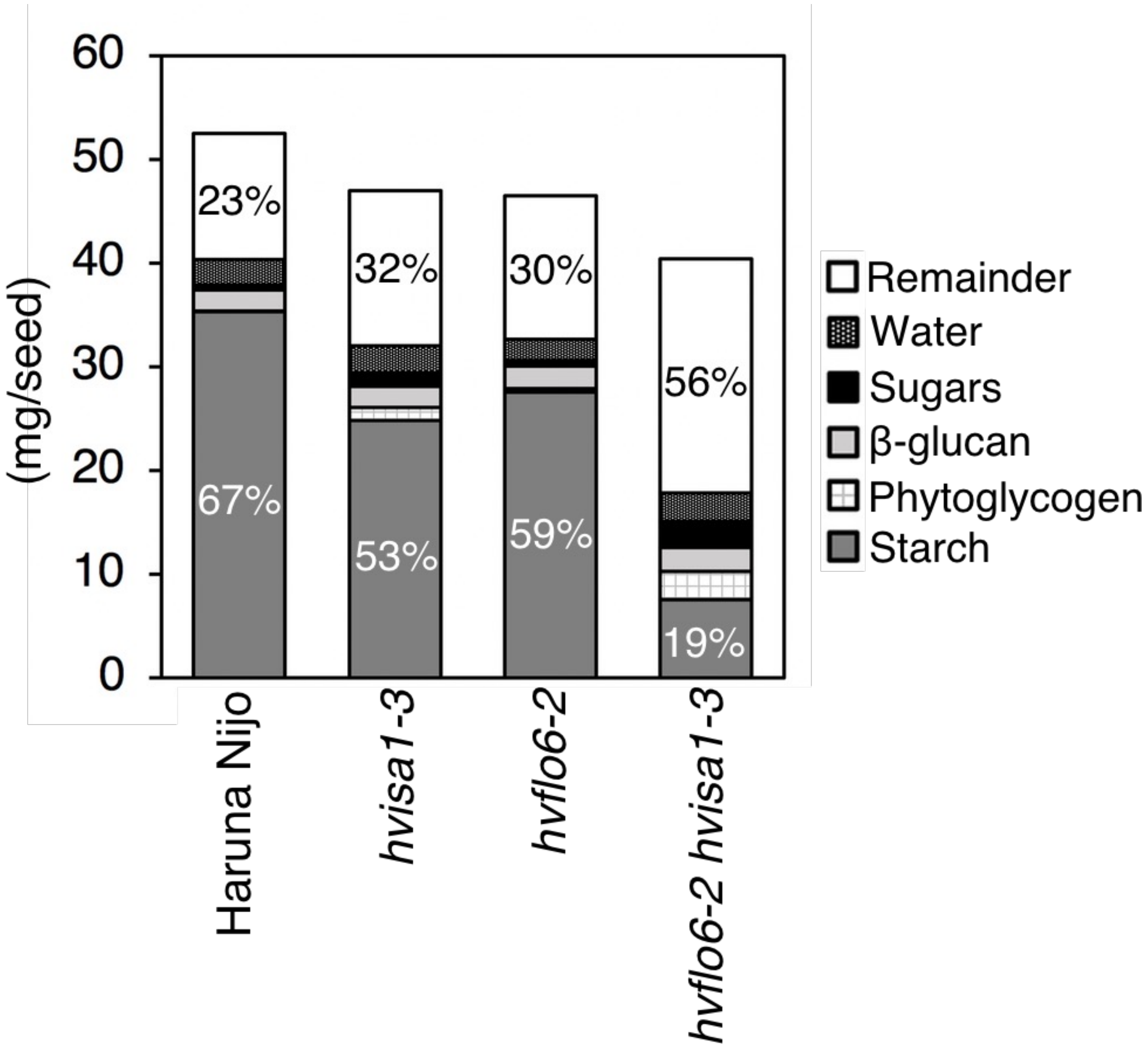

Supplementary Fig. 14 Stacked bar graph of the components in single mature grains. Data are average values from at least three biological replicates.
